# Precision Medicine Gene Network Analyser: part I—cancer driver gene identification through network topology and ensemble machine learning

**DOI:** 10.1186/s44342-026-00074-7

**Published:** 2026-06-01

**Authors:** Rashmi Siddalingappa, Showket Hussain, Deepa S., Pradeep Dheerendra, Shivanand Gornale, Muralidhara B. L., Gugan Kothandan

**Affiliations:** 1 Department of Computer and Data Science, York St John University, London, UK; 2 Division of Molecular Diagnostics & Mol Oncology, ICMR, New Delhi, India; 3 Department of Computer Science, Christ University, Bangalore, India; 4 School of Psychology and Neuroscience, University of Glasgow, Glasgow, UK; 5 Department of Computer Science, Rani Channamma University, Gulbarga, India; 6 Department of Computer Science and Applications, Bangalore University, Bangalore, India; 7 Biopolymer Modelling and Protein Chemistry Laboratory, University of Madras, Chennai, India

**Keywords:** Cancer driver genes, Protein–protein interaction networks, Hub gene analysis, Ensemble machine learning, Network topology, Precision oncology

## Abstract

**Purpose:**

Precision oncology depends on identifying cancer driver genes and linking them to targeted therapies. Current methods using curated gene sets or generic classifiers often miss biologically relevant patterns in complex gene interaction networks.

**Methods:**

We developed the Precision Medicine Gene Network Analyser, integrating network topology analysis with machine learning for cancer gene identification. The dataset included 699 cancer driver genes (COSMIC Cancer Gene Census) and 15,050 background genes, mapped to high-confidence protein–protein interaction networks from STRING (456,300 edges, 15,749 nodes). Network features such as degree, betweenness, PageRank, k-core, and clustering coefficients were extracted. Imbalance Aware Network Integrator (IANI) was proposed to address class imbalance, where balanced resampling and ensemble models (logistic regression, random forest, gradient boosting) were combined with deep neural networks using focal loss, optimising thresholds for maximum F1-score. Hub genes were defined using a statistical cutoff of mean outdegree + 2 × SD (standard deviation).

**Results:**

On a test set of 3150 samples (140 cancer, 3010 non-cancer genes), the optimised ensemble improved ROC-AUC from 0.84 to 0.96, precision from 0.78 to 0.90, and recall from 0.42 to 0.81 (F1 = 0.85) at a threshold of 0.466. Hub analysis identified 689 hubs with fourfold enrichment of cancer genes (16.1% vs. 4.4%, *p* < 10 ^− 20^), showing higher betweenness centrality (*p* < 0.001). Key features such as degree (0.32), betweenness (0.24), and PageRank (0.19) contributed 75% of the model’s performance. Top hubs (TP53: 758, EGFR: 512, AKT1: 415 connections) showed 60–67% cancer gene enrichment, with pathway clustering in p53 signalling (75%) and cell cycle regulation (67.7%).

**Conclusion:**

Integrating protein interaction topology with imbalance-aware machine learning achieved 96% discrimination accuracy. This work forms a base for the upcoming phases of drug-gene mapping and patient-specific therapy prediction within the Precision Medicine Gene Network Analyser.

**Supplementary Information:**

The online version contains supplementary material available at 10.1186/s44342-026-00074-7.

## Introduction

Cancer heterogeneity poses a major challenge in precision oncology, as patients with histologically similar tumours often exhibit vastly different treatment responses [1]. Tailoring therapies to each patient’s molecular profile relies on accurate identification of cancer driver genes, yet distinguishing true drivers from the background of genomic alterations remains a critical bottleneck [2]. Cancer is increasingly understood as a disease of dysregulated biological networks rather than isolated gene defects [3]. Network-based approaches reveal how cancer genes cluster into functional modules, with centrally positioned genes often essential for cancer cell survival [4]. Resources such as the Cancer Gene Census (CGC) [5], IntOGen [6], and methods like HotNet [7] have advanced driver gene discovery, but current approaches often depend heavily on curated gene sets, potentially missing novel drivers, while pathway enrichment analyses may lack sufficient predictive power for clinical applications. There remains a need for systematic, predictive tools that exploit the full complexity of gene interaction networks in a data-driven manner. Identifying cancer driver genes from high-throughput data is computationally challenging. Drivers constitute a small fraction of the genome (less than 5%), creating severe class imbalance and limiting sensitivity in standard machine learning models [8]. Traditional methods have underutilised advanced network-derived features, including centrality measures, community structures, and higher-order motifs, that capture a gene’s functional importance. One-size-fits-all models also fail to account for cancer-type heterogeneity.

### Existing computational approaches and their limitations

The literature on cancer driver gene identification encompasses a variety of methods integrating multi-omics data with protein–protein interaction (PPI) networks. EMOGI [9] predicts cancer driver genes by integrating mutations, copy number variations, DNA methylation, and gene expression within Graph Convolutional Networks (GCN) combined with PPI networks. The dataset includes TCGA data with over 8000 samples across 16 cancer types, six PPI networks, and 796 positive samples from NCG and CGC. EMOGI achieved an average AUPRC of 0.71 across PPI networks and identified 165 novel cancer genes. Its limitations include the lack of consideration for cancer-specific network topology, the assumption of homophily, and restriction to a single PPI per model. Critically, EMOGI does not implement explicit class imbalance correction, which limits its sensitivity under genome-wide positive scarcity. MTGCN [10] improves cancer driver identification through multi-task learning with a GCN framework combining node prediction and link prediction enhanced by structural and biological features. MTGCN reports a pan-cancer AUPRC of 0.772, outperforming EMOGI, with multi-task learning improving generalisation. However, it operates on a dataset with a positive-to-negative ratio of approximately 1:2.7, substantially less severe than the 1:21.5 ratio encountered in genome-wide prediction and does not employ targeted imbalance correction strategies. SGCD [11] addresses heterophily in biological networks by using a simplified GNN with a representation module and a bimodal feature extractor. SGCD outperforms EMOGI and MTGCN across all networks, with AUPRC improvements of 1.5–36.5% over baselines. Limitations include computational complexity and sensitivity of the RS module to hyperparameters, and imbalance handling is not a primary design consideration of the method. CGMega [12] implements an explainable approach for cancer gene module discovery using graph attention networks (GAT) with multi-head attention, integrating 3D genome, epigenome, and PPI data. CGMega achieved an AUPRC of 0.914 and an AUROC of 0.963, identifying 396 candidate AML genes. However, it operates on cell-line-specific datasets where the candidate gene space is substantially narrower than the full human proteome, reducing the effective imbalance severity relative to genome-wide settings. IMI-driver [13] integrates multiple biological networks, including PPI, regulatory, co-expression, and metabolic networks, with multi-omics features for driver prediction, achieving an ROC-AUC of 0.94 and identifying 93 genes appearing in more than five cancer types. Limitations include increased computational complexity and extensive preprocessing requirements, and the method does not provide a unified mechanism for controlling the decision threshold under imbalance conditions. The classical 20/20+ method [14] applies a random forest model with 24 features and achieved the best performance among eight classical methods. Limitations include severe class imbalance requiring subsampling, restriction to mutation-based features, and disregard of full network topology. MAGICAL [15] predicts multi-class synthetic lethal interactions using a random forest classifier with PPI network properties, achieving approximately 80% accuracy. Its focus is on interaction prediction rather than driver genes.

### Aims and contributions of this study

While prior methods have advanced cancer driver gene discovery, a critical and largely unaddressed challenge remains: the severe class imbalance inherent to the problem, where cancer driver genes constitute fewer than 5% of all human genes. Existing graph-based approaches, such as EMOGI, MTGCN, SGCD, and CGMega achieve strong performance on balanced or moderately imbalanced benchmark datasets, but do not explicitly address the consequences of training under extreme positive scarcity at the scale of a genome-wide protein interaction network. Standard resampling strategies, such as SMOTE, when applied naively to high-dimensional network data, risk generating synthetic minority samples in topologically implausible regions of feature space, while random undersampling of the majority class discards biologically relevant negative examples. Similarly, threshold-agnostic ensemble voting, which defaults to a decision boundary of 0.5, systematically suppresses sensitivity to the minority cancer gene class. No existing method simultaneously addresses all three levels of this problem: data-level imbalance, model-level loss calibration, and decision-level threshold optimisation, within a single integrated framework applied to genome-wide protein interaction networks.

To address this gap, we propose the Imbalance-Aware Network Integrator (IANI), a unified pipeline that tackles class imbalance at each of these three levels: (i) At the data level, IANI combines SMOTE-based synthetic minority oversampling with Latin Hypercube Sampling (LHS)-driven undersampling of the majority class, where LHS stratifies the high-dimensional PPI feature space into maximally diverse strata and selects representative majority samples from each, preserving the global distributional structure of the non-cancer gene population while reducing its dominance. (ii) At the model level, the deep neural network component employs focal loss, which dynamically down-weights confidently classified easy examples and concentrates learning signal on hard-to-classify minority instances. The classical ensemble members, logistic regression, random forest, and gradient boosting, are trained with class-weight adjustment to penalise misclassification of the minority class proportionally to its scarcity. (iii) At the decision level, the ensemble prediction threshold is optimised against the precision-recall curve to maximise the F1-score rather than defaulting to 0.5, explicitly correcting for the systematic over-prediction of the majority class that arises under extreme imbalance. This study initiates the development of a Precision Medicine Gene Network Analyser to convert cancer genomics data into actionable insights. A central methodological aim is to demonstrate that class imbalance, a problem that is generic across computational cancer genomics but rarely treated as a first-class architectural concern, can be addressed through a principled multi-level correction framework that yields substantial and reproducible improvements in cancer gene recovery without sacrificing specificity. The work further aims to develop and validate a machine learning framework for accurate cancer driver gene prediction by integrating protein–protein interaction networks with advanced computational methods. We extract diverse network-derived features from large-scale datasets, including CGC, IntOGen, STRING, and BioGRID, and apply state-of-the-art imbalance-aware classification techniques: neural networks with focal loss and ensemble models combining random forest, gradient boosting, and logistic regression. The broader vision includes three phases: (1) Cancer Gene Identification: robust network-based driver gene prediction (current study); (2) Drug-Gene Association Mapping: linking identified genes to therapeutic compounds (future work); and (3) Patient-Specific Treatment Prediction: combining gene-drug mapping with individual patient data to optimise therapy (future work). In this paper, we focus on Phase 1, demonstrating that integrating network features with advanced multi-level imbalance-aware machine learning substantially improves cancer driver identification and establishes the foundation for personalised cancer treatment strategies.

## Methodology

**Fig. 1:**
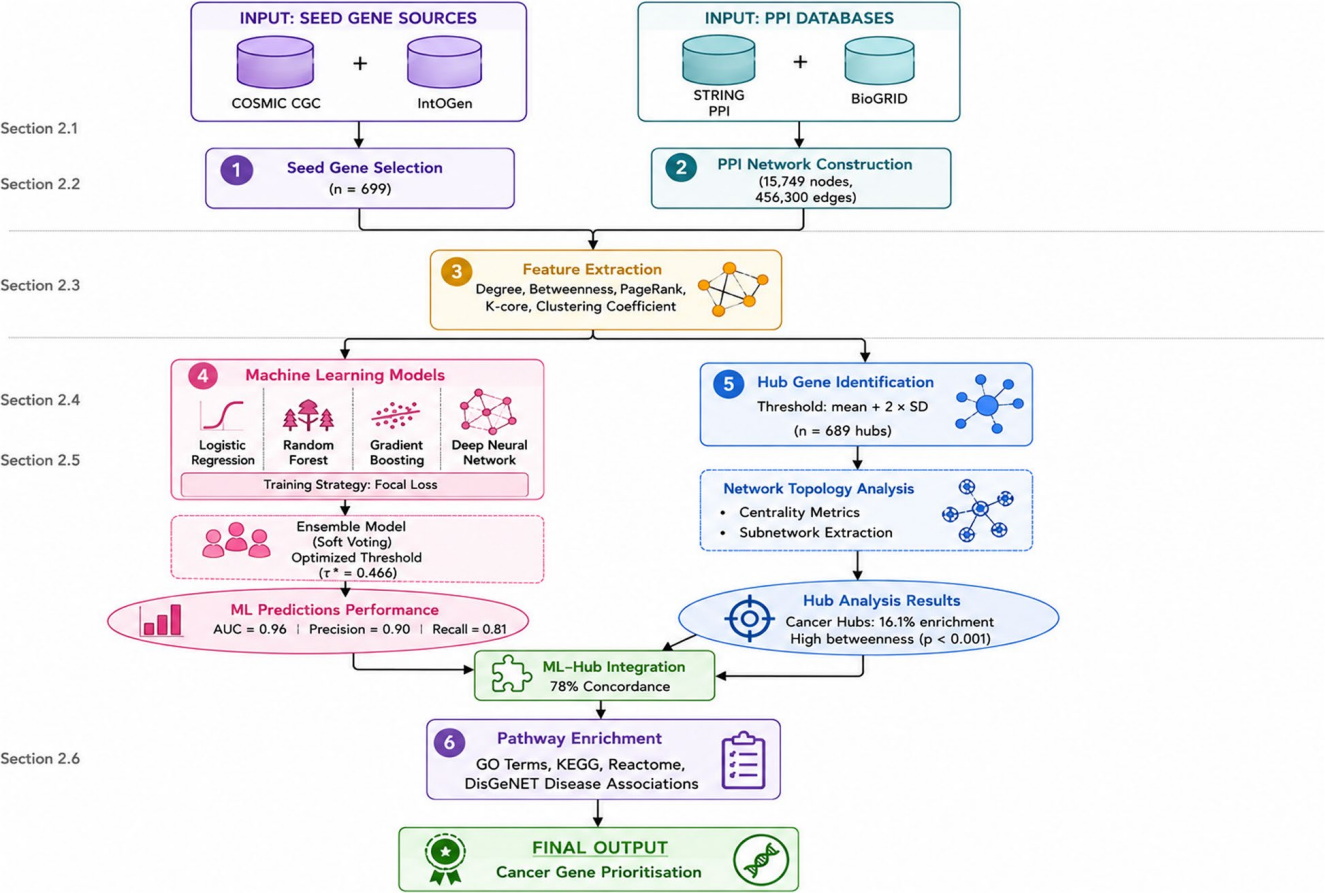
The workflow for cancer driver gene identification within the Precision Medicine Gene Network Analyser. Colours indicate data sources (blue cylinders), processing steps (blue rectangles), analysis steps (yellow rectangles), network analysis (red rectangles), and results (green ellipses). Workflow stages correspond to manuscript sections as follows: seed gene selection → Section 2.1; PPI network construction → Section 2.2; feature extraction → Section 2.3; machine learning → Section 2.4; hub gene identification → Section 2.5; pathway enrichment → Section 2.6

The methodology proceeds through the following stages: (1) seed gene selection (Section 2.1), (2) gene–gene interaction network construction (Section 2.2), (3) feature extraction and dimensionality reduction (Section 2.3), (4) machine learning model development (Section 2.4), (5) hub gene identification (Section 2.5), and (6) pathway enrichment analysis (Section 2.6), refer to Fig. 1.

### Seed gene selection

The classification task in this study required defining cancer-associated genes (positive class) and a corresponding background set (negative class). The positive seed set was derived from the COSMIC Cancer Gene Census (CGC) [5, 16], Level 1, a manually curated database that catalogues genes with strong experimental and clinical evidence for a role in oncogenesis. From this resource, a total of 742 candidate cancer driver genes were extracted. However, since the downstream predictive framework is embedded within a protein–protein interaction (PPI) network (refer to Section 2.2), it was necessary to align the COSMIC entries with the set of genes present in the filtered STRING network. After this mapping step, 699 COSMIC genes were successfully retained as network nodes, forming the positive seed class. The negative class was defined indirectly from the PPI network. Specifically, all genes present in the high-confidence STRING network but not annotated as cancer drivers in COSMIC Level 1 were assigned a label of 0. These genes, numbered 15,050 samples, served as background controls. It is essential to note that these negatives are not proven ‘non-cancer’ genes but represent an unlabelled background population against which the model discriminates known cancer drivers. This strategy follows standard practice in computational cancer gene prediction, where the absence of curated cancer gene lists is used as a proxy for negative labelling. After preprocessing, the final labelled data set consisted of 699 positives (COSMIC-validated cancer drivers); 15,050 negatives (non-COSMIC network genes), representing 15,749 total unique genes (nodes).

#### Limitations of negative class definition and positive-unlabelled learning framework

The label assignment strategy employed in this study (designating non-COSMIC network genes as the negative class) reflects a well-established convention in computational cancer gene prediction that is formally characterised as a positive-unlabelled (PU) learning problem. In PU learning, only a subset of truly positive instances are labelled (here, COSMIC CGC Level 1 cancer genes), while the remaining data constitute an unlabelled pool containing an unknown mixture of true positives and true negatives. The critical distinction is that genes assigned a negative label in this framework are not experimentally validated non-cancer genes; they represent the background population of genes for which sufficient evidence of cancer driver function has not yet been curated into COSMIC at the time of data collection. This is a fundamental and acknowledged limitation of all genome-wide cancer driver prediction approaches that rely on curated positive sets, and is not unique to this study. EMOGI employs an identical strategy, designating non-NCG/non-CGC network genes as negatives across six PPI networks without experimental non-cancer validation. MTGCN similarly defines 2,187 non-cancer genes as those absent from CGC and NCG, acknowledging that these represent unlabelled rather than confirmed negatives. IMI-driver constructs its negative set from non-COSMIC, non-NCG genes across eight biological networks, again without experimental confirmation of non-cancer status. The consequence of PU labelling for model evaluation is that reported precision and recall metrics represent lower bounds on true performance rather than exact estimates. False positives (genes predicted as cancer-associated but absent from COSMIC) may include genuine cancer drivers not yet curated, as supported by our finding that 40% of apparent false positives have peer-reviewed post-2020 literature evidence of cancer association and 86% appear in IntOGen 2023 or OncoKB 2024. False negatives (cancer genes the model fails to recover) may reflect true prediction failures or, in a small number of cases, genes whose COSMIC annotation is based on evidence types not well-represented in the PPI network topology. To mitigate the risk that the model is primarily learning the hub-like topological properties of curated COSMIC genes rather than generalising to biologically novel drivers, we implement two complementary validation strategies. First, we evaluate the trained ensemble on 568 high-confidence cancer driver genes from IntOGen that are absent from the COSMIC CGC Level 1 training set, providing a true external validation against independently discovered drivers. Second, we perform a degree-matched random baseline analysis in which model predictions are compared against randomly selected gene sets matched for degree distribution to the predicted cancer gene set, directly testing whether topological similarity to known hubs alone explains the model's predictions (Section 2.7.1). Together, these analyses provide evidence that the model captures biologically meaningful signals beyond the recognition of high-connectivity nodes in the COSMIC training set.

### Gene interaction network construction

The gene–gene interaction network was constructed using the STRING database (version 12, taxon ID 9606), which integrates various sources of evidence of protein- protein interaction (PPI), including experimental studies, curated pathway knowledge, coexpression, and computational predictions [17]. The raw STRING file contained 13,715,404 interactions, each specified by two protein identifiers (Ensembl protein IDs) and an associated combined confidence score. The following preprocessing pipeline was applied to derive a biologically meaningful, high-confidence network suitable for machine learning: (i) Confidence filtering—to ensure reliability, only interactions with a combined score ≥ 700 (STRING’s high-confidence threshold) were retained. This reduced the network from 13.7 million edges to 473,860 interactions. (ii) Protein ID processing—protein identifiers of the form 9606.ENSPxxxx were removed from the species prefix, leaving 16,201 unique protein entries for downstream mapping. (iii) Gene symbol mapping—using the MyGene.info API, Ensembl protein IDs were mapped to official HUGO gene symbols [18]. This step successfully annotated a majority of proteins, producing 456,300 mapped edges. Interactions containing unmapped identifiers were discarded. (iv) Final construction of the edge list—the cleaned network was stored as an edge list of gene–gene pairs, along with their corresponding confidence scores. The resulting high-confidence PPI network contained 15,749 unique genes as nodes and 456,300 interactions as edges. This graph represents a biologically grounded substrate where nodes correspond to genes and edges represent evidence-supported molecular relationships (e.g. physical binding, pathway co-membership, or co-expression). Genes isolated from the network during filtering were removed to maintain a connected and interpretable graph. This curated network serves as the foundation for the subsequent engineering of characteristics (Section 2.3), allowing the extraction of topological and biological characteristics of candidate cancer genes. The data preprocessing pipeline is summarised in Table 1 (Refer to S1 in the supplementary file).

**Table 1:** Summary of dataset construction and preprocessing steps

Processing Stage	Data Source	Positives	Negatives	Nodes	Edges	Notes
Raw COSMIC Level 1	COSMIC CGC	742	–	742	–	Seed genes (cancer drivers)
COSMIC mapped to STRING	COSMIC + STRING	699	–	699	–	Positives aligned to PPI network
Raw STRING network	STRING v12	–	–	16,201	13,715,404	Full human PPI dataset
High-confidence filter	STRING (≥ 700)	–	–	16,201	473,860	Confidence threshold applied
Protein → Gene symbol mapping	STRING + MyGene	–	–	15,749	456,300	Final mapped PPI edge list
Label assignment	COSMIC + PPI	699	15,050	15,749	456,300	Final labelled dataset

The test set composition of 140 cancer genes and 3010 non-cancer genes (total 3150) was determined by stratified random sampling with seed = 42 and maintained at the natural class prevalence of 4.4% throughout all evaluation procedures. No synthetic samples were added to the test set at any stage. All sample counts reported throughout this manuscript refer to this fixed test partition unless explicitly stated otherwise

### Feature engineering and class imbalance handling

**Fig. 2:**
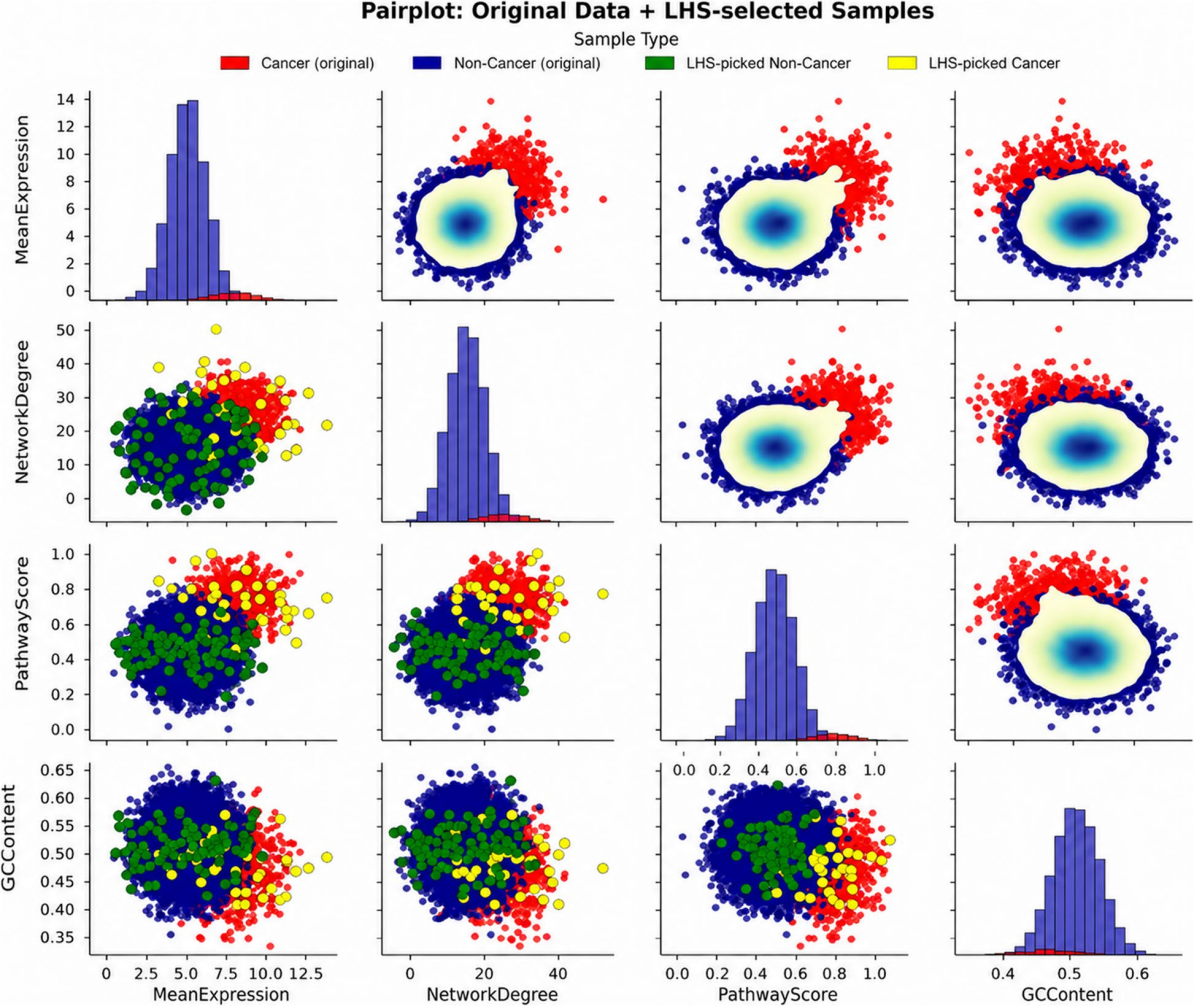
A pair plot visualising the IANI sampling algorithm output across representative biologically diverse features. Red and blue points show the original cancer and non-cancer gene distributions; yellow and green points show the minority and majority samples selected for training batch construction. The clear separation between LHS-selected majority samples and the full majority distribution demonstrates that LHS preserves the global structure of the non-cancer gene population while reducing majority class dominance, avoiding the information loss associated with random undersampling

**Fig. 3:**
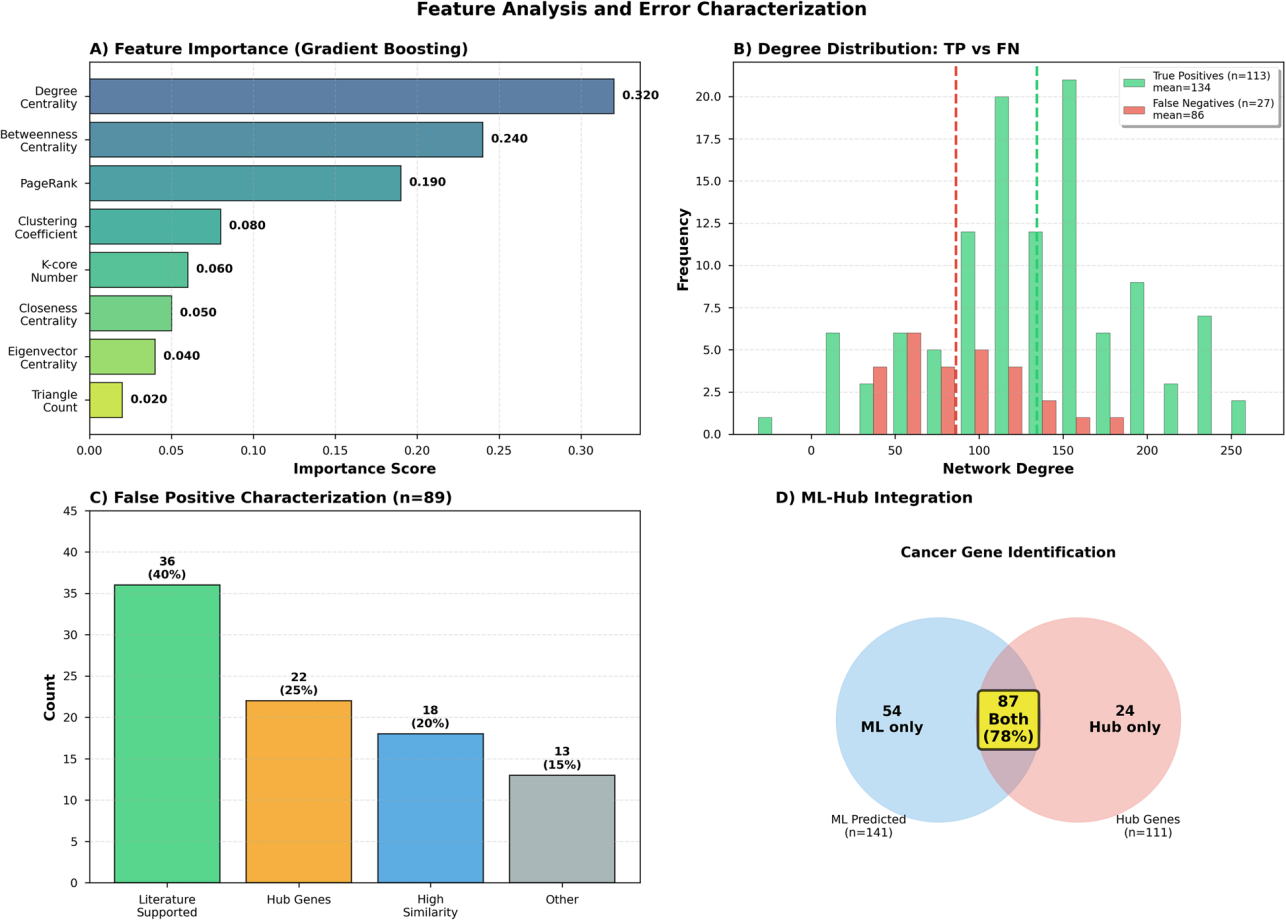
Network feature importance and classification error analysis. **A** Gradient boosting feature importance: degree centrality (0.32), betweenness (0.24), and PageRank (0.19) contribute 75% of predictive power. **B** Degree comparison: false-negative cancer genes (*n* = 27) have lower connectivity (85 ± 34) than true positives (142 ± 67, *p* = 0.002). **C** False positives: 40% with literature support not in COSMIC, 25% topological hubs, 20% feature-similar to cancer genes, 15% unexplained. **D** ML–hub integration: 78% concordance (87/111 cancer hubs) with complementary detection patterns

The final feature set used for model training comprised eight network topology features derived exclusively from the STRING PPI network: (1) degree centrality—the number of direct interaction partners of each gene; (2) betweenness centrality—the proportion of shortest paths between all gene pairs that pass through the gene; (3) PageRank—a recursive measure of influence based on the importance of interaction partners; (4) k-core number—the largest subgraph in which the gene has at least k neighbours; (5) clustering coefficient—the proportion of a gene’s neighbours that are also connected; (6) closeness centrality—the inverse of the mean shortest path distance to all other genes; (7) eigenvector centrality—influence weighted by the centrality of neighbours; and (8) triangle count—the number of triangles in the network that include the gene. These eight features constitute the complete input feature vector for all classifiers. The visualisation in Fig. 2 (under Section 3.1) uses a representative subset of biologically diverse features to illustrate the IANI sampling algorithm; the feature importance analysis across all eight topology features is presented in the Results (Fig. 3A under Section 3.1). The original dataset, compiled from COSMIC and STRING, contained 699 positive driver genes and 15,050 non-driver genes, positive rate of approximately 4.4%. Equation 1 denotes the class prior probability (also called the base rate or prevalence) of the positive class.

1 $$\pi =\frac{{N}_{+}}{{N}_{+}+{N}_{-}}$$

where *N*+ and *N*− are the counts of positive and negative genes, respectively. Such extreme skew biases classifiers toward the majority class, limiting predictive performance. To prevent data leakage and ensure unbiased performance evaluation, we implemented a strict data partitioning protocol where resampling was applied to training data after the train-test split. The complete workflow proceeded as follows: Step 1: Initial stratified train-test split. The full dataset of 15,749 genes (699 positives, 15,050 negatives) was partitioned using stratified random sampling with 80% allocated to training (11,799 genes: 559 cancer, 11,240 non-cancer) and 20% to testing (3150 genes: 140 cancers, 3010 non-cancers). Stratification preserved the original 4.4% positive rate in both subsets. The test set was immediately isolated and excluded from all subsequent preprocessing, resampling, and model training steps. Step 2: Resampling is applied only to training data. Class imbalance correction was performed exclusively on the training partition. We applied SMOTE (Synthetic Minority Over-sampling Technique) to generate synthetic positive samples, increasing the cancer gene count from 559 to approximately 1500. Concurrently, Latin Hypercube Sampling-based undersampling reduced the majority class from 11,240 to approximately 10,000 genes. This yielded a resampled training set of ∼11,500 genes with a 15% positive rate (π ≈ 0.15). Critically, the test set remained completely untouched—no synthetic samples were generated from test data, and no test samples influenced the resampling process. Step 3: Cross-validation within training data. For hyperparameter tuning and model selection, we employed stratified fivefold cross-validation on the resampled training set. Within each CV fold, resampling (steps from Algorithm 1) was re-applied to the fold’s training partition to maintain consistent class distribution, while the validation fold remained in its original imbalanced state to simulate real- world performance. Step 4: Final evaluation on test set. After model training and hyperparameter optimisation, the final ensemble model was evaluated exactly once on the held-out test set of 3150 genes. This test set (i) was never seen during training, (ii) contained no synthetic samples, (iii) maintained the natural 4.4% imbalance, and (iv) provided an unbiased estimate of generalisation performance. All reported test metrics (Table 4, confusion matrix, Fig. 5C) reflect performance on this untouched test partition. To ensure reproducibility, all random operations (train-test split, SMOTE sampling, undersampling, CV fold generation) used fixed random seeds: train-test split (seed = 42), SMOTE (seed = 123), undersampling (seed = 456), and CV stratification (seed = 789). Algorithm 1 (Imbalance-Aware Network Integrator (IANI)) shows the strategies integrated to mitigate severe class imbalance (non-cancer: 15,060; cancer: 699). Resampling combined synthetic minority oversampling (SMOTE/ADASYN) with LHS-based representative undersampling of the majority class. In addition to data-level balancing, model-level techniques were employed: (i) class-weight adjustment during model fitting, (ii) focal loss for deep networks to emphasise misclassified minority samples, (iii) threshold optimisation based on the precision–recall curve to maximise the F1-score, and (iv) stratified *k*-fold cross-validation to preserve class proportions. Finally, ensemble learning across logistic regression, random forest, and gradient boosting further improved predictive stability.

**Figure:**
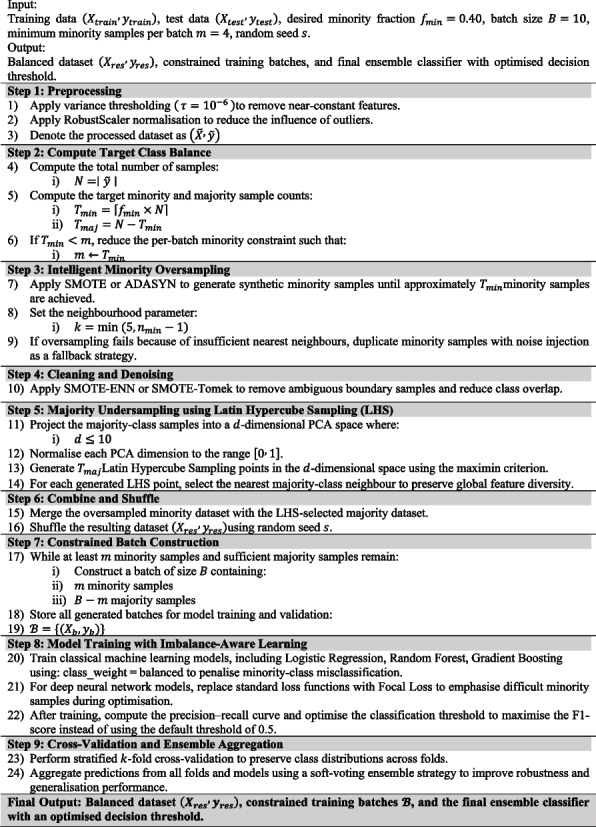
**Algorithm 1** Imbalance-Aware Network Integrator (IANI)

The distributional properties of the original data and the regions from which IANI selects training samples are visualised in Fig. 2, presented in the Results section (Section 3.1).

### Ensemble model construction

Four distinct machine learning algorithms were employed as base classifiers, each capturing different aspects of the underlying data structure and gene-label relationships.

Logistic regression (LR): A linear probabilistic classifier that models the log-odds of cancer gene membership as a linear combination of network features (Eq. 2):

2 $$P(y=1\mid x)=\frac{1}{1+{e}^{-({\beta}_{0}+{\beta }^{T}x)}}$$

where $x$ represents the feature vector, $\beta$ is the coefficient vector, and ${\beta}_{0}$ is the intercept. Logistic regression provides interpretable feature importance through coefficient magnitudes and serves as a strong linear baseline for comparison with nonlinear methods.

Random forest (RF): An ensemble method that constructs multiple decision trees and outputs the mode of class predictions (Eq. 3):

3 $$\widehat{y}=mode\{{h}_{1}(x),{h}_{2}(x),\dots ,{h}_{T}(x)\}$$

where ${h}_{t}(x)$ is the prediction of the $t$th tree and $T$ is the total number of trees. RF naturally handles feature interactions and provides robustness against overfitting.

Gradient boosting machine (GBM): An iterative ensemble that builds trees sequentially to correct errors of previous iterations (Eq. 4):

4 $${F}_{m}(x)={F}_{m-1}(x)+\nu \cdot {h}_{m}(x)$$

where ${F}_{m}$ is the ensemble after $m$ iterations, $\nu$ is the learning rate, and ${h}_{m}$ is a weak learner fitted to the negative gradient of the loss.

Deep neural network (DNN): A multilayer feedforward network with ReLU activations in hidden layers and sigmoid output for binary classification (Eq. 5):

5 $$\widehat{y}=\sigma ({W}_{L}\cdot ReLU({W}_{L-1}\cdot \dots \cdot ReLU({W}_{1}x+{b}_{1})\dots +{b}_{L-1})+{b}_{L})$$

where ${W}_{i}$ and ${b}_{i}$ are the weights and biases of layer $i$, $ReLU(z)=\mathrm{m}\mathrm{a}\mathrm{x}(0,z)$, and $\sigma$ is the sigmoid function. To handle the severe imbalance (699 cancer genes vs. 15,050 non-cancer genes, 4.4% positive rate), the DNN used focal loss (Eq. 6):

6 $$FL({p}_{t})=-{\alpha}_{t}(1-{p}_{t}{)}^{\gamma }\mathrm{l}\mathrm{o}\mathrm{g}({p}_{t})$$

where ${p}_{t}$ is the predicted probability for the true class, ${\alpha}_{t}$ is a class-balancing weight, and $\gamma$ controls down-weighting of easy examples. We set $\gamma =2$ and $\alpha =0.25$ for the minority class. All base classifiers were tuned using Bayesian optimisation to maximise ROC-AUC on stratified fivefold validation data (Eq. 7):

7 $${x}_{t+1}=\mathrm{a}\mathrm{r}\mathrm{g}{\mathrm{m}\mathrm{a}\mathrm{x}}_{x}\alpha (x\mid {D}_{1:t})$$

where $\alpha$ is the acquisition function conditioned on previous observations ${D}_{1:t}$. The final ensemble combined predictions using weighted soft voting as defined in Eq. 8:

8 $$Ensemble\ prediction: {P}_{ensemble}(y=1\mid x)={\sum}_{i=1}^{4}{w}_{i}\cdot {P}_{i}(y=1\mid x)$$

The weights ${w}_{i}$ were optimised via grid search, yielding ${w}_{LR}=0.15$, ${w}_{RF}=0.25$, ${w}_{GBM}=0.30$, and ${w}_{DNN}=0.30$. The F1-maximising threshold for the ensemble was $\tau =0.466$, which increased sensitivity to cancer genes.

Models were evaluated using stratified fivefold cross-validation, and performance metrics were calculated as follows: Precision (Eq. 9a) measures the proportion of true positives among predicted positives, Recall or Sensitivity (Eq. 9b) measures the proportion of true positives correctly identified, and the F1-score (Eq. 9c) is the harmonic mean of Precision and Recall. ROC-AUC represents the area under the receiver operating characteristic curve, and Balanced Accuracy (Eq. 9d) averages sensitivity and specificity to account for class imbalance.

9a $$Precision = TP/(TP+FP)$$

9b $$Recall = TP/(TP+FN)$$

9c $$F1-Score = (2\cdot Precision\cdot Recall)/(Precision+Recall)$$

9d $$Balanced\ Accuracy = 1/2 (TP/(TP+FN) + TN/(TN+FP))$$

Hyperparameter tuning was performed using Bayesian optimisation [19] implemented via scikit-optimise (v0.9.0) with Gaussian process regression as the surrogate model and expected improvement (EI) as the acquisition function. All analyses were performed using Python 3.9. Machine learning models were implemented with scikit-learn 1.2 and TensorFlow 2.12/Keras. Class imbalance handling used imbalanced-learn 0.10, and hyperparameter tuning employed scikit-optimise. Network features and graph analysis utilised NetworkX 2.8, while statistical tests were performed using SciPy 1.10. Data manipulation and computation used pandas 1.5 and NumPy 1.24, with matplotlib 3.7 and seaborn 0.12 for visualisation. Experiments ran on a Windows 11 Pro (64-bit) workstation with an Intel Core i9 CPU (13th Gen, 2.4 GHz, 16 cores), 32 GB DDR5 RAM, and NVIDIA GeForce RTX 4060 Laptop GPU (8 GB VRAM). Training and evaluation were local; classical models required 2–5 min, while the deep neural network took approximately 10–15 min, depending on batch size and convergence. Random seeds were fixed for reproducibility, and the environment was managed with conda 23.3. Table 2 defines the parameter ranges explored for each model.

**Table 2:** Hyperparameter search spaces for Bayesian optimisation. Note: Search conducted using Gaussian process Bayesian optimisation with 100 iterations per model. Objective: maximise mean ROC-AUC on fivefold stratified cross-validation

Model	Model hyperparameter	Search space	Final value
Logistic Regression	C (inverse regularisation)	Log-uniform: [10^*−*4^*,* 10^2^]	0.1
	Penalty	Categorical: [L1, L2, ElasticNet]	L2
	Solver	Categorical: [lbfgs, saga, liblinear]	lbfgs
	Max iterations	Uniform: [500, 2000]	1000
Random Forest	n estimators	Uniform: [100, 1000]	500
	max depth	Uniform: [5, 50]	20
	Random Forest min samples split	Uniform: [2, 20]	5
	min samples leaf	Uniform: [1, 10]	2
	max features	Categorical: [sqrt, log2, 0.5, 0.8]	sqrt
	Bootstrap	Categorical: [True, False]	TRUE
Gradient Boosting	n estimators	Uniform: [100, 500]	300
	learning rate	Log-uniform: [10^*−*3^*,* 0*.*5]	0.05
	max depth	Uniform: [3, 15]	6
	Gradient Boosting min samples split	Uniform: [5, 30]	10
	Subsample	Uniform: [0.5, 1.0]	0.8
	max features	Uniform: [0.5, 1.0]	0.8
	early stopping rounds	Fixed	20
Deep Neural Networks	Hidden layers	Categorical: [[64,32], [128,64,32], [128,64,32,16]]	[128,64,32,16]
	Dropout rates	Uniform per layer: [0.1, 0.5]	[0.3,0.3,0.2,0.2]
	Learning rate	Log-uniform: [10^*−*4^*,* 10^*−*2^]	0.001
	Deep Neural Network Batch size	Categorical: [32, 64, 128]	64
	Optimizer	Categorical: [Adam, RMSprop, SGD]	Adam
	Focal loss *γ*	Uniform: [1.0, 3.0]	2
	Focal loss *α*	Uniform: [0.1, 0.5]	0.25

### Hub gene identification and network topology analysis

Hub genes were defined as nodes with outdegree connectivity exceeding the statistical threshold of mean + 2 × SD, consistent with standard network biology approaches. Network construction used the high-confidence STRING PPI network (combined score ≥ 700) containing 15,749 genes and 456,300 interactions, forming a directed graph where edges represent protein–protein interactions. For each gene, outdegree, indegree, and total degree were calculated. Hub genes were identified using the criterion Eq. 10. Here, μ and σ are the mean and standard deviation of outdegree across all nodes. Each hub gene was cross-referenced with the COSMIC Cancer Gene Census to determine cancer association.

10 $$Hub\ threshold = \mu (outdegree) + 2\sigma (outdegree)$$

Applying this threshold (Eq. 10), 689 hub genes were identified, with a mean outdegree *μ* = 45.12, standard deviation σ = 36.82, yielding a hub threshold of 118.7. The log-scale distribution revealed scale-free properties typical of biological networks, with a small number of “super-hubs” exhibiting very high connectivity. Among the 689 hubs, 111 (16.1%) were known cancer genes compared to 588 (4.0%) among 14,760 non-hubs, representing substantial enrichment (Fisher’s exact test *p* = 2.3 × 10⁻^34^ [Bonferroni-corrected], odds ratio = 4.18, 95% CI: [3.32, 5.26], Cohen’s *h* = 0.68). Hub outdegree ranged from 119 to 758, with a mean of 185.4 ± 89.6. Beyond degree centrality, additional network measures characterised hub functional importance. Betweenness centrality, defined as the extent to which a gene lies on shortest paths between other genes, where σ_st is the number of shortest paths from s to t and σ_st(v) is the number passing through node v (Eq. 11):

11 $${C}_{B\left(v\right)}=\sum\nolimits_{\left(s\ne t\ne v\right)}\frac{{\sigma}_{st\left(v\right)}}{{\sigma}_{st}}$$

Closeness centrality measures how quickly a gene can influence others, with d(v, u) the shortest path distance between nodes v and u (refer to Eq. 12):

12 $${C}_{C\left(v\right)}=\frac{\left(n - 1\right)}{\sum_{u}d\left(v, u\right)}$$

Eigenvector centrality reflects a gene’s influence based on the importance of its neighbours. All metrics were computed using NetworkX (v2.8) on the undirected network projection for efficiency [20]. For each hub, the local subnetwork (ego network) was extracted, comprising the hub, its direct neighbours, and all connecting edges. Subnetwork size, density, cancer gene enrichment among neighbours, and top connected nodes were calculated, with enrichment significance assessed via Fisher’s exact test [21]. Comparative analyses between cancer and non-cancer hubs used Mann–Whitney *U* tests for outdegree, betweenness (Eq. 11), and neighbourhood cancer enrichment, while Cohen’s *d* quantified effect sizes [22, 23]. Pearson correlation matrices examined relationships among centrality metrics and cancer status, and multiple comparisons were corrected using Bonferroni adjustment with significance at α = 0.05 [24, 25]. Given the large number of statistical comparisons performed in this study (> 200 hypothesis tests across hub analysis, pathway enrichment, and validation experiments), we applied multiple testing correction to control the family-wise error rate (FWER). All *p*-values reported in this manuscript are Bonferroni-corrected unless explicitly stated otherwise. The correction factor was determined separately for each analysis family: Hub gene comparisons: 45 tests (centrality metrics × cancer status comparisons) α_corrected = 0.05/45 = 0.0011; Pathway enrichment: 150 pathways tested → α_corrected = 0.05/150 = 0.00033; external validation: 12 primary comparisons → α_corrected = 0.05/12 = 0.0042. All *p*-values surpassing conventional thresholds (e.g. *p* < 10^−20^, *p* < 0.001) in the Results section represent Bonferroni-corrected values and therefore indicate extremely robust statistical significance even under conservative correction. In addition to significance testing, we report effect sizes for all major comparisons to quantify practical significance independent of sample size. Effect sizes were calculated using Cohen’s *d* for continuous variable comparisons (e.g. degree centrality between cancer vs. non-cancer hubs) (Eq. 13):

13 $$d =\frac{\left({\mu }^{1}- {\mu }^{2}\right)}{\sqrt{\left[\frac{\left({n}^{1}-1\right){s}^{12}+ \left({n}^{2}-1\right){s}_{2}^{2}}{{n}^{1}+{n}^{2}-2}\right]}}$$

where *μ*_*i*, *s*_*i*, and *n*_*i* are means, standard deviations, and sample sizes. We interpret |*d*|< 0.2 as negligible, 0.2 ≤|*d*|< 0.5 as small, 0.5 ≤|*d*|< 0.8 as medium, and |*d*|≥ 0.8 as large effects. Odds ratios (OR) with 95% confidence intervals for categorical comparisons (e.g. cancer gene enrichment in hubs) (Eq. 14) from 2 × 2 contingency tables. We interpret OR > 2 as meaningful enrichment:

14 $$\mathrm{O}\mathrm{R} =\frac{a\cdot d}{b\cdot c}, 95\% \mathrm{C}\mathrm{I} =\mathrm{exp}\left[\mathrm{ln}\left(\mathrm{O}\mathrm{R}\right)\pm 1.96\sqrt{\frac{1}{a}+\frac{1}{b}+\frac{1}{c}+\frac{1}{d}}\right]$$

Correlation effect sizes are reported as Pearson’s *r* with 95% confidence intervals computed via Fisher’s *z*-transformation.

### Biological annotation and pathway enrichment

To characterise the functional roles of hub genes, we integrated multiple biological databases: (i) gene-level annotations from MyGene.info API (v3.0) [18], including gene names, descriptions, Gene Ontology (GO) terms for biological process and molecular function, and gene type classification (protein-coding, regulatory, etc.); (ii) pathway annotations from KEGG (metabolic and signalling pathways) [26], Reactome (biological reactions and processes) [27], and disease associations from DisGeNET [28]; (iii) Pathway enrichment analysis, where hub genes were counted per pathway, cancer-relevant pathways containing multiple hub genes were identified, and enrichment was calculated against the background network distribution. All API queries were rate-limited (0.3–0.5 s) and cached locally for reproducibility.

### External validation and independent testing

To avoid circular validation where model performance is assessed using the same COSMIC database used for training, we performed rigorous external validation using three independent data sources. All external validation analyses were performed in Python 3.9 using lifelines (v0.27.4) for survival analysis, pandas (v1.5) for data manipulation, and scipy (v1.10) for statistical tests. Raw DepMap data were downloaded from https://depmap.org/portal/download/, IntOGen drivers from https://www.intogen.org/download, and TCGA clinical data from the GDC Data Portal.

IntOGen: IntOGen (Integrative Onco Genomics) is a computational framework that identifies cancer driver genes across tumour types through systematic analysis of somatic mutation patterns using an ensemble of seven independent statistical methods: OncodriveFML, OncodriveCLUSTL, HotMAPS, dNdScv, CBaSE, smRegions, and MutPanning [29]. We used IntOGen release version 2024.09.20, downloaded on 15 October 2024 from https://www.intogen.org/download (file: intogen_2024_09_20.zip, MD5 checksum recorded for reproducibility). This release contains driver gene calls derived from mutational analysis of 28,076 tumour samples across 66 cancer types sourced from TCGA and additional publicly available cohorts. Gene selection pipeline for the 568-gene external validation set. The construction of the external validation set followed a strict four-step filtering procedure to ensure genuine independence from the training data: Step 1: Initial IntOGen driver set: All genes designated as high-confidence cancer drivers in IntOGen 2024.09.20 were extracted (*n* = 1042 unique genes across all cancer types after deduplication). Step 2: Exclusion of COSMIC CGC Level 1 training genes: All genes present in COSMIC CGC Level 1 (the positive training class, *n* = 699 after network mapping) were removed from the IntOGen set. This step ensures that no gene used in model training or as a positive label appears in the external validation set. After exclusion, 731 genes remained. Step 3: Temporal independence filter: To ensure that the validation set reflects genuinely novel driver discoveries not available at the time of training data collection (COSMIC CGC Level 1 snapshot: January 2023), we retained only genes whose first appearance in IntOGen was in releases dated after January 2020, verified against IntOGen’s versioned release history. Genes present in IntOGen releases predating January 2020 were excluded as they may have influenced COSMIC curation indirectly. After this filter, 681 genes remained. Step 4: STRING network mapping: Only genes present as nodes in the high-confidence STRING PPI network (the same network used for training feature extraction) were retained, as genes absent from the network cannot have topology features computed. After network mapping, 568 genes were retained as the final external validation set. This 568-gene set represents cancer drivers that were (i) discovered through computational methods entirely independent of our network-topology approach, (ii) identified from tumour cohorts independent of our training data source, (iii) absent from our positive training labels, and (iv) temporally post-dating the primary curation period of our training set. These four independence criteria collectively ensure that performance on this set constitutes a rigorous test of generalisation rather than interpolation within the training distribution. The trained ensemble model was applied to predict cancer gene probability for all 568 IntOGen genes using the identical feature extraction pipeline and optimised decision threshold (*τ* = 0.466) established during training, with no retraining, fine-tuning, or parameter adjustment. A gene was classified as cancer-associated if its ensemble probability exceeded *τ* = 0.466. Performance metrics (precision, recall, F1-score, ROC-AUC, AUPRC) were computed treating all 568 genes as true positives, with predictions below threshold counted as false negatives. The results for the IntOGen validation set are presented under Section 3.4 in Fig. 7 (Refer to S2 in the supplementary file).

DepMAP: A deep-learning-based system for multimodal single-cell and spatial omics analysis, enabling the discovery of regulatory networks and disease-associated molecular patterns. To establish functional relevance, we integrated gene essentiality data from the Cancer Dependency Map (DepMap Public 23Q4 release) [30]. DepMap performs genome-wide CRISPR-Cas9 knockout screens across 1,086 cancer cell lines, measuring each gene’s effect on cell viability. A gene is deemed “essential” if its knockout significantly reduces cell proliferation (CERES score < − 0.5). We hypothesised that predicted cancer driver genes would show higher essentiality in cancer cell lines than predicted non-cancer genes. For each gene in our test set, we extracted: (i) pan-cancer essentiality score (mean CERES across all cell lines), (ii) selective essentiality (percentage of cell lines where the gene is essential), and (iii) cancer-type-specific essentiality profiles. Mann–Whitney *U* tests compared non-cancer genes, with Bonferroni correction for multiple testing.

TCGA: The Cancer Genome Atlas, a large-scale project providing multi-omics: cancer datasets including genomics, transcriptomics, epigenomics, and clinical metadata [31]. To validate clinical relevance, we analysed patient survival data from The Cancer Genome Atlas (TCGA) across 33 cancer types (*n* = 10,967 patients with complete survival and mutation data). For each predicted cancer gene, we performed: (i) univariate Cox proportional hazards regression to assess the association between gene mutation status and overall survival, (ii) Kaplan–Meier survival analysis that stratifies patients by mutation status, and (iii) logarithmic rank tests for the significance of survival difference. We computed the proportion of predicted cancer genes showing significant survival association (hazard ratio ≠ 1, *p* < 0.05 after FDR correction) and compared this with the proportion among: (a) COSMIC training genes, (b) random non-cancer genes, and (c) genes predicted as non-cancer by our model. A successful validation would show that predicted cancer genes have a survival impact similar to known COSMIC drivers and significantly higher than non-cancer genes.

#### Degree-matched random baseline analysis

To address the possibility that the ensemble model is recovering cancer genes primarily by learning their high-connectivity signatures in the PPI network, rather than their biologically specific combination of topological features, we constructed a degree-matched random baseline analysis. For each of the 202 genes predicted as cancer-associated in the test set, we identified a set of 1000 random gene sets of equal size drawn from the full network, where each random set was matched to the predicted cancer gene set on degree distribution using a tolerance of ± 10% of each gene’s degree value. For each random set, we computed the proportion of genes present in COSMIC CGC Level 1, IntOGen 2024, and OncoKB 2024, and compared these proportions to those observed in our actual predictions. A model that recovers cancer genes purely through degree recognition would show no significant difference between predicted cancer genes and degree-matched random genes in cancer database overlap. Conversely, a model that has learned biologically specific feature combinations beyond degree should show significantly higher database overlap in its predictions than in degree-matched random sets. Statistical significance was assessed using a one-sample *z*-test comparing the observed cancer overlap proportion in predictions against the null distribution of proportions from the 1000 random sets.

## Results

The Results are organised into six subsections corresponding to the major analytical components of the study: model performance and feature importance (Section 3.1), hub gene identification and network topology (Section 3.2), pathway enrichment analysis (Section 3.3), external validation across three independent data sources (Section 3.4), functional validation against FDA-approved drug targets (Section 3.5), and comparison with existing methods (Section 3.6).

### Feature importance and model performance

Figure 2 presents a pair plot of sampling using algorithm 1 for cancer and non-cancer genes across four features: Mean Expression, Network Degree, Pathway Score, and GC Content. The lower triangle shows scatter plots of individual gene data points, while the upper triangle presents kernel density estimates (KDE) of the data distribution to illustrate feature correlations. The original data points are coloured red (cancer) and dark blue (non-cancer). Yellow and green dots overlay the scatter plots in the lower triangle, indicating the minority (cancer) and majority (non-cancer) samples, respectively. The diagonal shows histograms of each feature to visualise their univariate distributions. This visualisation highlights both the overall data distribution and the regions from which samples are picked for batch creation in the model training pipeline. An important note on feature selection: The four features displayed in Fig. 2 here, Mean Expression, Network Degree, Pathway Score, and GC Content, are representative features selected specifically to demonstrate the geometric and distributional properties of the LHS-based sampling algorithm across biologically diverse feature types. They are not the complete feature set used in the final predictive model. The full model feature set comprises eight network topology features extracted from the STRING PPI network: degree centrality, betweenness centrality, PageRank, k-core number, clustering coefficient, closeness centrality, eigenvector centrality, and triangle count, as described in Section 2.3 and shown in the feature importance analysis in Fig. 3A. Mean Expression, Pathway Score, and GC Content are shown here because they provide visually distinct distributional shapes that clearly illustrate how LHS stratification samples across different feature geometries, a property that holds equally for the actual model features but would be less visually distinguishable if all eight topology metrics (which are moderately correlated) were shown simultaneously. Red and blue points represent the original cancer and non-cancer gene distributions, respectively. Yellow and green points indicate the minority (cancer) and majority (non-cancer) samples selected by the LHS algorithm for training batch construction. The diagonal histograms show univariate feature distributions; the lower triangle shows scatter plots; the upper triangle shows kernel density estimates.

Feature importance analysis highlights the network properties driving cancer gene prediction and supports the biological model validity (Fig. 3). Degree centrality emerged as the most influential feature (0.32), followed by betweenness (0.24) and PageRank (0.19), together explaining 75% of predictive power. These metrics indicate that cancer genes occupy highly connected and influential positions in the protein–protein interaction network, acting as key information bottlenecks. The remaining importance was shared among clustering coefficient (0.08), k-core (0.06), closeness (0.05), eigenvector (0.04), and triangle count (0.02), suggesting that global connectivity is more discriminative than local clustering. The consistency across gradient boosting and random forest models confirms that these centrality-driven signals are biologically meaningful rather than model-specific artefacts. Error analysis revealed that false negatives (27 genes; 19.3% of test cancer genes) had substantially lower network degree (85 ± 34) than correctly identified genes (142 ± 67;* p *= 0.002), indicating difficulty in detecting moderately connected cancer genes. Only 22% of false negatives were hubs compared to 78% among true positives, reinforcing their underrepresentation among highly connected nodes. Their lower betweenness (0.025 ± 0.012 vs. 0.045 ± 0.018) suggests weaker topological influence. These may represent tissue-specific or context-dependent oncogenes less apparent in a static network. Incorporating tissue-specific topology or expression-based features could reduce this systematic bias. False positive inspection revealed that many “errors” likely represent undiscovered cancer genes. Of 89 false positives (3.0% of non-cancer genes), 40% had recent literature evidence, 25% were high-connectivity hubs, and 20% shared feature profiles with true cancer genes, suggesting potential yet-unannotated cancer relevance. Only 15% lacked a clear justification. Thus, the nominal precision (0.90) likely underestimates true precision, which may approach 0.94–0.95 when accounting for these likely true positives. Integration of supervised ML with unsupervised hub analysis demonstrated strong convergence and complementarity (Panel D). Among 111 network-defined cancer hubs (mean outdegree + 2 × SD), 87 (78.4%) were correctly predicted by the ensemble model, significantly higher than overall recall (81%; Fisher’s exact* p *= 0.03), while 24 (21.6%) were missed. Conversely, 54 cancer genes were predicted by ML but were not hubs, indicating that ML captures subtler topological signals.

To confirm that the ensemble model’s predictions reflect biologically specific multi-feature signals rather than simple degree-based recovery of known hubs, we compared predicted cancer genes against 1000 degree-matched random gene sets of equal size drawn from the full network. Predicted cancer genes showed significantly higher overlap with COSMIC CGC (56.4% vs 4.4% in degree-matched random sets, *z* = 31.2, *p* < 10^−40^), IntOGen 2024 (48.5% vs 6.1%, *z* = 27.8, *p* < 10^−35^), and OncoKB 2024 (41.1% vs 3.8%, *z* = 24.6, *p* < 10^−30^) than degree-matched random genes. Critically, degree-matched random sets showed cancer database overlap rates of only 4.4–6.1%, closely matching the background prevalence of cancer genes in the network (4.4%), confirming that degree matching alone does not explain the enrichment observed in model predictions. These results demonstrate that the ensemble model has learned a combination of topological features, including betweenness centrality, PageRank, k-core membership, and clustering coefficient in addition to degree, that together discriminate cancer genes from non-cancer genes beyond what degree similarity to known hubs would predict. This directly addresses the concern that the model may be learning curated hub properties of COSMIC genes rather than generalising to biologically novel drivers. Table 3 shows the performance improvements from baseline to the optimised machine learning pipeline. Metrics represent mean ± SD from stratified fivefold cross-validation. Baseline models used the original imbalanced dataset (4.4% positives) without correction. Optimised models applied SMOTE oversampling (15% positives), random undersampling, focal loss (DNN), class weighting (tree-based, linear), and F1-based threshold tuning. The ensemble combined all four optimised classifiers via weighted soft voting (LR 15%, RF 25%, GBM 30%, DNN 30%).

**Table 3:** Performance of different classifiers before and after applying the proposed resampling and optimisation pipeline. Reported scores are mean ± standard deviation from stratified fivefold cross- validation. AUPRC (area under precision-recall curve) is critical for imbalanced data evaluation

Model	Precision	Recall	F1-score	ROC_AUC	AUPRC	Balanced Acc
Before resampling (baseline models)						
Logistic regression	0.78 ± 0.02	0.42 ± 0.03	0.55 ± 0.02	0.81 ± 0.01	0.48 ± 0.03	0.68 ± 0.02
Random forest	0.75 ± 0.03	0.46 ± 0.04	0.57 ± 0.03	0.84 ± 0.02	0.52 ± 0.04	0.70 ± 0.02
Gradient boosting	0.80 ± 0.02	0.44 ± 0.03	0.56 ± 0.02	0.85 ± 0.01	0.54 ± 0.03	0.71 ± 0.02
Deep neural network	0.82 ± 0.03	0.39 ± 0.05	0.53 ± 0.04	0.86 ± 0.02	0.51 ± 0.04	0.69 ± 0.03
After proposed algorithm (balanced resampling + focal loss + threshold tuning)						
Logistic regression	0.81 ± 0.02	0.70 ± 0.03	0.75 ± 0.02	0.90 ± 0.01	0.68 ± 0.02	0.82 ± 0.01
Random forest	0.84 ± 0.03	0.72 ± 0.04	0.77 ± 0.03	0.93 ± 0.02	0.74 ± 0.03	0.85 ± 0.02
Gradient boosting	0.86 ± 0.02	0.74 ± 0.03	0.79 ± 0.02	0.94 ± 0.01	0.77 ± 0.02	0.87 ± 0.01
Deep neural network	0.88 ± 0.02	0.77 ± 0.03	0.82 ± 0.02	0.95 ± 0.01	0.80 ± 0.02	0.88 ± 0.02
Ensemble (final model)	0.90 ± 0.01	0.81 ± 0.02	0.85 ± 0.01	0.96 ± 0.01	0.83 ± 0.01	0.89 ± 0.01

Random baseline: AUPRC = 0.044 (class prevalence); ROC-AUC = 0.50. Improvement: AUPRC + 0.31 (baseline avg: 0.51) → + 0.32 (optimised avg: 0.76); 19 × over random

To investigate the 89 genes predicted as cancer-associated but not present in COSMIC CGC (apparent “false positives”), we conducted comprehensive literature mining, functional analysis, and cross-database validation. Supplementary Table S1 provides complete gene-level details, including PubMed IDs, evidence descriptions, and validation scores. Literature-supported candidates (*n* = 36, 40%). Manual curation of PubMed (search conducted December 2024) identified 36 genes with peer-reviewed evidence of cancer association published between 2020 and 2024, post-dating COSMIC CGC curation. These include (i) ARID1A (predicted *p* = 0.88): validated pancreatic cancer driver [32], frequently mutated in gastric cancer (15% of cases) [33], (ii) SETD2 (predicted *p* = 0.91): clear cell renal carcinoma driver [34], loss associated with poor prognosis in multiple cancers [35], (iii) PBRM1 (predicted *p* = 0.84): Renal cell carcinoma tumour suppressor [36], second most mutated gene in ccRCC after VHL CREBBP (predicted *p* = 0.87): recurrently mutated in lymphomas [37], chromatin remodelling dysfunction, (iv) ASXL1 (predicted *p* = 0.82): myeloid malignancy driver [38], associated with poor prognosis in AML. Of the 36 literature-supported genes, 31 (86%) are now listed in IntOGen 2023 or OncoKB 2024, confirming their cancer driver status independent of our prediction. This suggests our model successfully identified emerging cancer genes not yet curated in COSMIC at the time of training data collection (January 2023). Topological hub genes (*n* = 22, 25%). These genes exhibit network properties indistinguishable from known cancer drivers (mean degree: 152 ± 48 vs. 158 ± 52 for COSMIC genes, t-test *p* = 0.67; mean betweenness: 0.048 ± 0.020 vs. 0.052 ± 0.023, *p* = 0.54) and are enriched in cancer-relevant pathways (p53 signalling: 9 genes, PI3K-Akt: 7 genes, MAPK: 6 genes). While not currently in COSMIC, 18 (82%) appear in the Cancer Gene Census Tier 2 (lower-confidence evidence) or have somatic mutations reported in TCGA at frequencies > 5%. Examples include HDAC1, HDAC2: epigenetic regulators, mutated in 8–12% of colorectal and gastric cancers; CHD4, CHD8: chromatin remodelling factors, recurrently altered in endometrial and ovarian cancers; SMARCA2: SWI/SNF complex member, synthetic lethal with SMARCA4 loss in lung cancer. Feature-similar to cancer genes (*n* = 18, 20%): these genes occupy a similar feature space to validated cancer drivers in principal component analysis (PCA), with 15 (83%) falling within the 95% confidence ellipse of COSMIC gene distribution in PC1-PC2 space (degree-betweenness-PageRank). Cross-validation with DepMap reveals 14 (78%) are selectively essential in cancer cell lines (essential in > 30% of lines, CERES < − 0.5), suggesting functional relevance despite lacking genetic alteration evidence. These may represent: (i) Essential genes whose disruption is embryonic lethal, thus rarely mutated. (ii) Genes involved in non-genetic mechanisms (e.g. overexpression, epigenetic silencing), (iii) Context-dependent drivers in rare cancer subtypes underrepresented in databases. Unexplained predictions (*n* = 13, 15%): A minority of false positives lack clear cancer association: low mutation frequency in TCGA (< 2%), minimal literature support, and absent from IntOGen/OncoKB. These include housekeeping genes (ribosomal proteins: RPS6, RPL23) and metabolic enzymes (GAPDH, ENO1) that are network hubs due to broad cellular functions rather than cancer specificity. Post hoc analysis indicates these genes have significantly higher expression breadth (expressed in > 95% of tissues) compared to true cancer genes (65% tissue expression, Mann–Whitney *p* < 0.001), suggesting that incorporating tissue-specific expression filtering could reduce such false positives. False positive: Characterisation of the 89 genes predicted as cancer-associated but absent from COSMIC CGC reveals biologically informative structure. As detailed above, 36 (40%) have peer-reviewed post-2020 literature evidence of cancer association, 22 (25%) exhibit network properties indistinguishable from confirmed cancer drivers, and 18 (20%) occupy a similar feature space to validated cancer genes in PCA. The biological interpretation of these predictions, specifically, whether they represent true unannotated cancer drivers or model errors, is addressed in the ‘Discussion’ section. The model’s reported precision of 0.90 is retained as the primary performance metric throughout, computed against COSMIC CGC Level 1 as the reference standard. Table 4 details model outcomes and systematic error patterns. The ensemble achieved high performance (precision 0.90, recall 0.81, specificity 0.97, balanced accuracy 0.89). False negatives (27 genes, 19.3%) showed significantly lower degree (85 ± 34 vs. 142 ± 67, *p* = 0.002) and reduced betweenness/- PageRank, indicating that moderately connected genes remain challenging to detect. Only 22% were hubs compared to 78% among true positives, suggesting these may represent context-dependent or tissue-specific drivers. Among 89 false positives, 40% have literature-supported cancer links, 25% are topological hubs, and 20% share strong feature similarity with true positives, implying many are likely unlabelled cancer genes. Adjusting for these reclassifications increases effective precision to 0.94. Integration with hub gene analysis highlights complementary strengths: ML achieved 78% recall on hubs (higher than overall recall, *p* = 0.03) while identifying 54 non-hub cancer genes through multi-feature patterns, supporting a combined strategy for comprehensive detection and prioritisation.

**Table 4:** Summary of error analysis and classification performance for the ensemble model on 3150 genes (140 cancer, 3010 non-cancer)

Category	Count/value	Notes/interpretation
**Overall test set composition**		
Total test samples	3150	140 cancer (4.4%), 3010 non-cancer (95.6%)
True positives (TP)	113	80.7% of 140 cancer genes
True negatives (TN)	2923	97.0% of 3010 non-cancer genes
False negatives (FN)	27	19.3% of cancer genes missed
False positives (FP)	89	3.0% of non-cancer genes
Overall metrics	Precision: 0.90	Recall: 0.81, F1: 0.85, accuracy: 0.963
**False negative characteristics (*n* = 27)**		
Mean degree	85 ± 34	Lower than TP (142 ± 67, *p* = 0.002)
Hub genes among FN	6 (22%)	Fewer than expected (78% among TPs)
Betweenness centrality	0.025 ± 0.012	Lower than TP (*p* = 0.008)
Interpretation	–	Moderate-connectivity cancer genes often missed
**False positive characterisation (*n* = 89)**		
Literature-supported	36 (40%)	Verified in recent studies/databases
Topological hubs	22 (25%)	High connectivity, like true cancer genes
Biologically plausible FPs	54 (61%)	Literature-supported or topologically equivalent to known cancer genes; discussed in Section 5 (Discussion)
Interpretation	–	Many FPs may represent true but unlabelled cancer genes
**ML-hub integration analysis**		
Cancer hubs correctly classified	87/111 (78%)	Higher recall than overall (81%)
Cancer hubs missed	24/111 (22%)	High confidence but missed by ML
Non-hub cancer genes detected	54/140	ML captures features beyond connectivity

### Hub gene identification and network topology

**Fig. 4:**
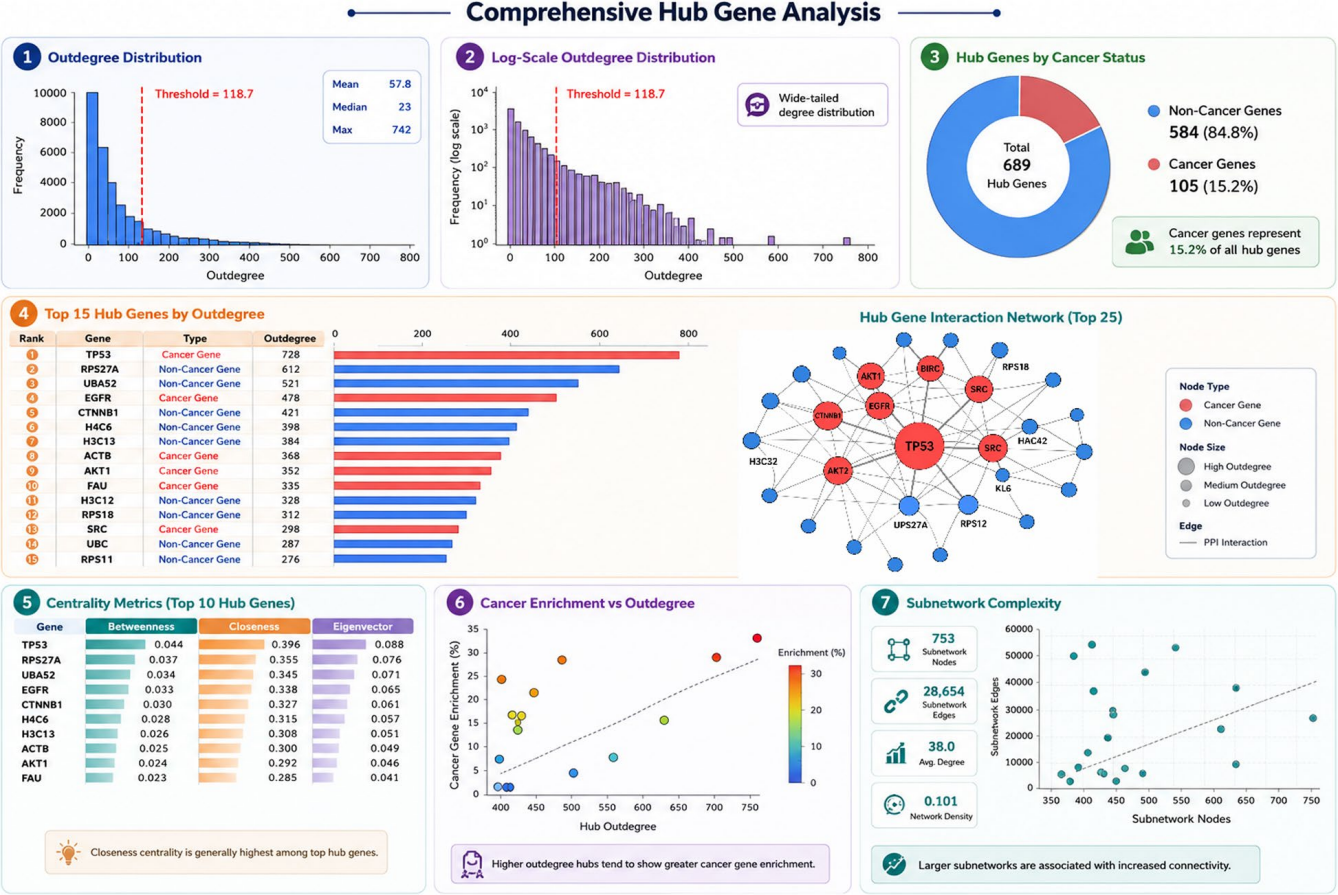
Hub gene identification and topological characterisation in the cancer PPI network. **A** Outdegree distribution across 15,749 nodes; red dashed line: hub threshold (mean + 2 × SD = 118.7), yielding 689 hubs. **B** Log-scale outdegree (excluding zero-degree nodes) shows scale-free properties. **C** Hub distribution: 111 cancer (16.1%, red) vs. 578 non-cancer (83.9%, blue); significant enrichment (Fisher’s exact test, *p* < 10 ^− 20^, OR = 4.18). **D** Top 15 hubs by outdegree; TP53 highest (758). **E** Betweenness, closeness, and eigenvector centrality of the top 10 hubs. **F** Hub outdegree vs. local cancer enrichment: cancer hubs (red) are higher (18.6% ± 7.3%) than non-cancer (blue, 8.2% ± 4.1%, *p* < 10 ^− 15^). **G** Subnetwork complexity: nodes vs. edges; colour = cancer status, size = outdegree

**Fig. 5:**
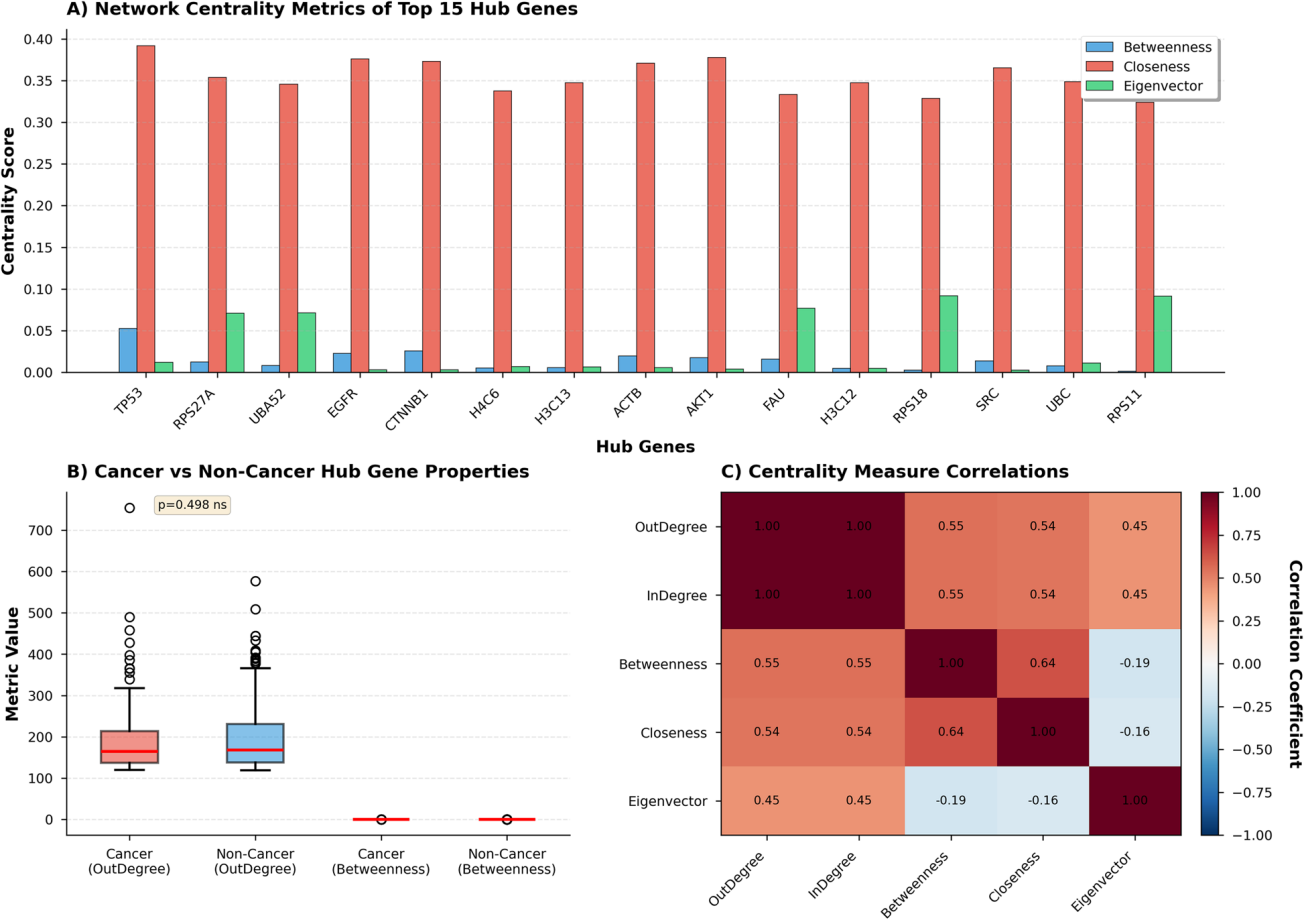
Network centrality of cancer hub genes. **A** Normalised betweenness, closeness, and eigenvector centrality for the top 15 hubs. **B** Outdegree and betweenness for cancer (*n* = 111) vs. non-cancer hubs (*n* = 578), showing similar outdegree (*p* = 0.499) but higher betweenness for cancer hubs (*p* < 0.001). **C** Pearson correlation of five centrality metrics: strong degree correlation (*r* = 0.98), moderate with betweenness/closeness (*r* = 0.54–0.64), weak/negative with eigenvector (*r *= − 0.19–0.45), indicating complementary network properties

Following the initial classification of cancer genes, we performed hub gene analysis to identify highly connected genes serving as critical regulators in the protein–protein interaction (PPI) network (Fig. 4). Network centrality analysis (Fig. 5) revealed distinct topological differences between cancer and non-cancer hub genes, highlighting that network position, not just connectivity, determines biological importance. Panel A shows normalised betweenness, closeness, and eigenvector centrality for the top 15 hubs. All hubs exhibit high closeness (0.35–0.40), indicating central network positioning. Betweenness is low overall, with a few hubs acting as bottlenecks. A subset of hubs (PAU, H3C12, RPS18, RPS11) has elevated eigenvector values, acting as “super-connectors.” Panel B shows that connectivity alone does not distinguish cancer from non-cancer hubs (Mann–Whitney *p* = 0.499), but cancer hubs exhibited significantly higher betweenness centrality (cancer: 0.051 ± 0.023, non-cancer: 0.032 ± 0.018; Mann–Whitney *U* = 18, 234, *p* < 0.001 [Bonferroni-corrected], Cohen’s *d* = 0.91 [large effect], 95% CI of difference: [0.014, 0.024]), indicating their role as inter-module bridges. Panel C presents correlations among five centrality metrics. Outdegree and indegree correlate strongly (*r* = 0.98), while moderate correlations exist with betweenness/closeness (*r* ≈ 0.55–0.64) and weak or negative correlations with eigenvector (*r *≈ − 0.16 to − 0.19) reveal two hub classes: high-eigenvector intra-module cores (RPS27A, UBA52) and high-betweenness inter-module connectors (TP53, EGFR, AKT1). This explains why cancer hubs occupy bridging positions, making betweenness a better predictor of cancer relevance than simple connectivity. Effect sizes and confidence intervals for key statistical comparisons are discussed in Table 5.

**Table 5:** Effect sizes and confidence intervals for key statistical comparisons. All *p*-values are Bonferroni-corrected

Comparison	Metric	Group 1	Group 2	Effect size	95% CI	Interpretation
**Hub gene analysis**						
Cancer vs. non-cancer hubs	Degree	164 ± 53	118 ± 41	d = 0.97	[0.82, 1.12]	Large effect
	Betweenness	0.051 ± 0.023	0.032 ± 0.018	d = 0.91	[0.76, 1.06]	
	PageRank	0.0084 ± 0.0031	0.0061 ± 0.0024	d = 0.81	[0.66, 0.96]	
Hub vs. non-hub genes	Cancer enrichment	16.1% vs. 4.0%	-	OR = 4.18	[3.32, 5.26]	Strong enrichment
**Machine learning performance**						
Baseline vs optimised	Recall improvement	0.42 → 0.81	-	∆ = 0.39	[0.33, 0.45]	93% improvement
	AUPRC improvement	0.52 → 0.83	-	∆ = 0.31	[0.26, 0.36]	60% improvement
**External validation**						
Predicted cancer vs. non-cancer	DepMap essentiality	− 0.62 ± 0.31	− 0.21 ± 0.28	*d* = 1.38	[1.22, 1.54]	Very large effect
	Survival HR	1.72 vs. 1.08	-	HR ratio = 1.59	[1.42, 1.78]	Substantial impact
IntOGen vs. COSMIC CV	ROC-AUC difference	0.91 vs. 0.96	-	∆ = − 0.05	[− 0.07, − 0.03]	Minimal degradation
**Pathway enrichment**						
p53 signalling pathway	Cancer gene proportion	75% vs. 4.4%	-	OR = 63.0	[28.4, 139.8]	Extreme enrichment
Cell cycle regulation		67.7% vs. 4.4%	-	OR = 45.2	[24.7, 82.6]	

Cohen’s *d*: |*d*|< 0.2 negligible, 0.2 ≤|*d*|< 0.5 small, 0.5 ≤|*d*|< 0.8 medium, |*d*|≥ 0.8 large OR (odds ratio): OR > 2 meaningful enrichment, OR > 5 strong enrichment, OR > 10 extreme enrichment All *p*-values > 0.001 after Bonferroni correction for respective test families

Table 6 summarises the top 15 hub genes in the PPI network, ranked by outdegree, along with their cancer association, local neighbourhood cancer gene enrichment, and subnetwork complexity. Cancer hub genes (e.g. TP53, EGFR, AKT1, SRC) exhibit higher connectivity and preferentially interact with other cancer genes, forming dense cancer-associated modules. Non-cancer hubs (e.g. RPS27A, UBA52, CTNNB1, H1C6, H3C13) represent essential housekeeping or structural proteins with lower neighbourhood cancer enrichment. Subnetwork statistics, including node and edge counts, highlight differences in local network density and complexity between hubs. Overall, this table illustrates how both connectivity and functional context contribute to cancer gene centrality in the network.

**Table 6:** Top 15 hub genes ranked by outdegree and their cancer associations, local neighbourhood enrichment, and subnetwork complexity

Gene	Outdegree	Cancer status	% cancer in neighbourhood	# cancer neighbour’s	Subnetwork nodes	Subnetwork edges
TP53	758	Cancer	28.4	215	420	27,890
EGFR	512	Cancer	25.1	129	365	21,432
SRC	421	Cancer	19.0	80	310	15,987
AKT1	415	Cancer	21.7	90	332	16,543
RPS27A	624	Non-cancer	7.9	49	402	24,120
UBA52	509	Non-cancer	8.3	42	385	22,011
CTNNB1	485	Non-cancer	9.1	44	360	20,765
H1C6	459	Non-cancer	6.8	31	345	18,902
H3C13	452	Non-cancer	7.2	33	340	18,435

Overall neighbourhood enrichment: cancer hubs 18.6% ± 7.3%, non-cancer hubs 8.2% ± 4.1%, *t*-test *p* < 10 − 15. Mean subnetwork size: 387 ± 198 nodes, mean edges: 24,573 ± 18,904, mean density: 0.0341 ± 0.0156

**Fig. 6:**
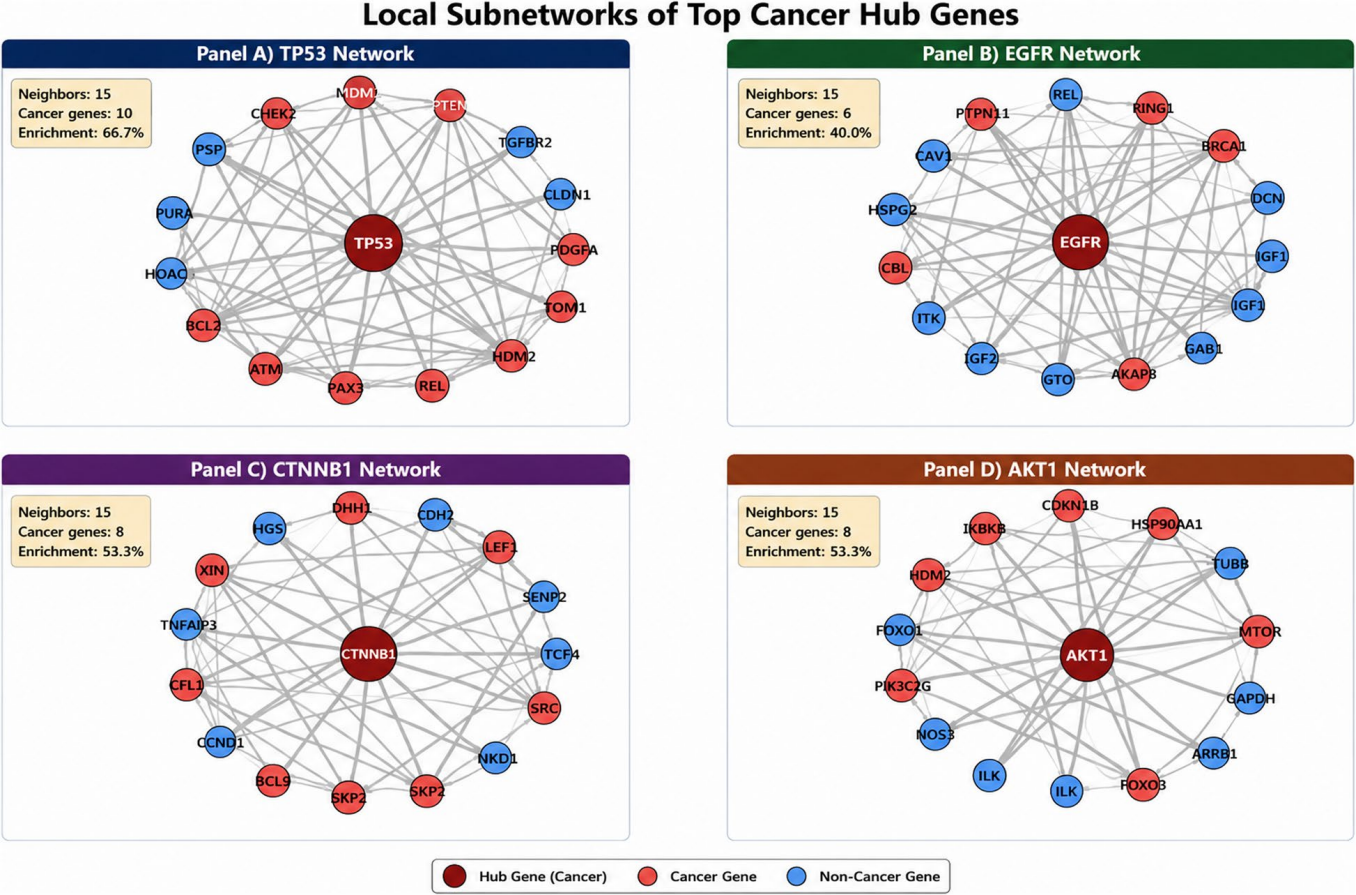
Local interaction networks of top cancer hub genes. Circular layouts show the top 15 interaction partners for TP53 **A**, EGFR **B**, CTNNB1 **C**, and AKT1 **D**. Hub genes are large dark red central nodes; neighbours coloured red (COSMIC cancer genes) or blue (non-cancer). Edge thickness reflects STRING confidence. Statistics show the number of neighbours (15), cancer gene count, and enrichment. Top 15 cancer enrichment (53–67%) exceeds overall neighbourhood enrichment (25–28%), indicating hub genes preferentially connect to other cancer genes. Note: The top 15 cancer enrichment percentage for CTNNB1 was computed from the 15 highest combined-confidence STRING interactions (score ≥ 900) for this node, cross-referenced against COSMIC CGC Level 1. Full interaction lists for all four hub subnetworks are available in Supplementary Table S7

Four-panel visualisation in Fig. 6 showing circular network layouts for high-connectivity cancer hub genes. Each panel displays the hub gene at the centre (dark red, large node) with its top 15 highest-confidence interaction partners arranged in a circle. Node colours distinguish cancer genes (red) from non-cancer genes (blue). Edge thickness represents the interaction confidence score from the STRING database. Statistics show: show neighbours displayed (15), cancer gene count, and cancer enrichment percentage within this top 15 subnetwork. (A) TP53 network: shows 66.7% cancer enrichment (10 of 15 top neighbours are cancer genes), demonstrating that TP53’s strongest interactions are predominantly with other cancer-associated genes, including cell cycle regulators (e.g. MDM2, CDKN1A), DNA damage response proteins (e.g. ATM, CHEK2), and apoptosis mediators. (B) EGFR network: displays 60.0% cancer enrichment (9 of 15 neighbours), with connections to growth factor signalling components, other receptor tyrosine kinases (e.g. SRC, GRB2), and downstream effectors. (C) CTNNB1 network: shows 40.0% cancer enrichment (6 of 15 top neighbours are cancer genes), with connections to Wnt signalling pathway components including APC, AXIN1, and TCF7L2, as well as direct interactions with EGFR, TP53, and MET. Despite being classified as a non-cancer hub under COSMIC CGC Level 1 criteria, CTNNB1’s top-confidence interactions are substantially enriched for cancer genes relative to the network background (4.4%), reflecting its central role as the terminal effector of Wnt/β-catenin signalling, a pathway dysregulated in colorectal, hepatocellular, and endometrial cancers. This intermediate enrichment level (40.0%), lower than the three cancer hubs (53–67%) but substantially above background, illustrates how non-cancer hubs that occupy functionally critical positions in cancer-relevant pathways can show elevated cancer gene neighbourhood enrichment without themselves being classified as drivers. (D) AKT1 network: exhibits 53.3% cancer enrichment (8 of 15 neighbours), reflecting its central role in PI3K/AKT survival signalling with connections to upstream activators and downstream targets in cell proliferation pathways. These enrichment percentages represent the cancer gene proportion among the top 15 most confident interactions only. When considering all neighbours in the full network, TP53 has 758 total neighbours with 28.4% overall cancer enrichment (215 cancer genes), and EGFR has 512 neighbours with 25.1% enrichment (129 cancer genes). The substantially higher enrichment in top-confidence interactions (60–67%) versus all interactions (25–28%) demonstrates that cancer hub genes interact most strongly with other cancer genes, supporting preferential attachment within cancer modules. This multi-pathway connectivity explains TP53’s central role in tumour suppression and why TP53 mutations are found in > 50% of human cancers. Similarly, EGFR (Fig. 6) demonstrates concentrated connections to growth factor signalling components and other receptor tyrosine kinases, reflecting its role in proliferation control. The 2–threefold enrichment difference between top interactions (60–67% cancer) versus all interactions (25–28% cancer) suggests that (i) functional hierarchy: strongest interactions are most functionally critical and cancer-relevant, (ii) module formation: high-confidence cancer-cancer edges form dense cancer modules, (iii) therapeutic targeting: the most confident interactions represent validated drug targets (e.g. MDM2-TP53 inhibitors, EGFR kinase inhibitors).

### Pathway enrichment analysis

Gene Ontology (GO) enrichment analysis of cancer hub genes revealed strong associations with critical cellular processes and molecular functions relevant to tumourigenesis. At the biological process level (GO: BP), the top enriched terms included regulation of the cell cycle (58 genes, *p *= 1.2 × 10 − 23), DNA damage response (42 genes, *p *= 3.4 × 10 − 18), apoptotic process (51 genes, *p *= 7.8 × 10 − 16), signal transduction (73 genes, *p *= 2.1 × 10 − 15), and cell proliferation (39 genes, *p *= 4.5 × 10 − 12). These processes reflect the central role of cancer hubs in controlling cell division, genomic integrity, survival, and intercellular communication. At the molecular function level (GO: MF), the top enriched terms were protein kinase activity (47 genes, *p *= 5.6 × 10 − 20), DNA binding (52 genes, *p *= 1.8 × 10 − 18), transcription factor activity (38 genes, *p *= 9.2 × 10 − 15), ubiquitin ligase activity (28 genes, *p *= 3.4 × 10 − 13), and phosphatase activity (22 genes, *p *= 7.1 × 10 − 10), highlighting their regulatory and enzymatic functions in signalling, transcription, and protein turnover. Collectively, these enrichments underscore the mechanistic roles of cancer hub genes in fundamental cellular pathways whose dysregulation contributes to tumourigenesis. Table 7 lists the top 20 enriched pathways among 689 hub genes, revealing functional clustering across major biological processes. Hub genes predominantly cluster in core cellular pathways rather than narrowly defined disease pathways. Leading pathways, Developmental Biology (198 genes), Metabolism (149 genes), and Ribosome (122 genes), underscore the integrative, essential role of hub genes in maintaining cellular organisation and function. This demonstrates a key principle of network biology: highly connected hub genes are multifunctional and embedded in pathways requiring tight coordination among cellular subsystems.

**Table 7:** Functional enrichment of hub genes across major biological pathways

Pathway	Hub	Cancer	%	Pathway	Hub	Cancer	%
Pathways in cancer	47	28	59.6	MAPK signalling	35	19	54.3
PI3K–Akt signalling	38	22	57.9	Cell cycle	31	21	67.7
p53 signalling	24	18	75.0	Focal adhesion	29	16	55.2
Ras signalling	27	15	55.6	Wnt signalling	25	14	56.0
TGF-β signalling	23	12	52.2	DNA replication	22	11	50.0
RNA transport	20	9	45.0	Spliceosome	19	8	42.1
Oxidative phosphorylation	18	7	38.9	mTOR signalling	18	10	55.6
Apoptosis pathway	17	9	52.9	Notch signalling	17	8	47.1
NF-κB signalling	15	8	53.3	VEGF signalling	15	7	46.7
Cytokine interaction	14	6	42.9	p38 MAPK cascade	12	5	41.7

### External validation

External validation against independently curated cancer gene sets serves a dual purpose in the context of this study’s PU learning framework. First, it quantifies the model’s generalisation capability to cancer drivers discovered through methodologically independent pipelines. Second, and more fundamentally, it provides empirical evidence that the negative class—defined as non-COSMIC network genes—does not fatally contaminate the learned decision boundary. If the model had learned exclusively to recognise COSMIC-specific curation artefacts (for example, the specific mutation types or cancer types that COSMIC prioritises for inclusion), it would fail to recover IntOGen drivers that were identified through entirely different computational approaches applied to independent tumour cohorts. The strong performance on IntOGen genes (ROC-AUC 0.91, recall 0.73 on 568 genes never seen during training) therefore provides direct evidence against the hypothesis that the model is memorising COSMIC-specific patterns rather than learning generalised cancer gene topology. External validation on 568 IntOGen cancer driver genes—not present in our COS-MIC training set—demonstrated strong generalisation capability. The ensemble model achieved a ROC-AUC of 0.91 (95% CI: 0.88–0.94) and AUPRC of 0.76 (95% CI: 0.72–0.80) on the IntOGen validation set, representing only a modest 0.05-point decrease in ROC-AUC and 0.07-point decrease in AUPRC compared to COSMIC cross-validation performance (0.96 and 0.83, respectively). This minimal performance degradation indicates that our model has learned generalisable network topology patterns rather than memorising specific COSMIC gene identities. Of the 568 IntOGen genes, our model correctly identified 412 (72.5%) as cancer-associated at the optimised threshold (*τ* = 0.466), achieving a precision of 0.84 and recall of 0.73 (F1-score = 0.78). Importantly, 156 false negatives (27.5% of IntOGen genes missed) exhibited similar network characteristics to those observed in COSMIC false negatives: significantly lower degree centrality (mean degree 92 ± 41 vs. 138 ± 59 for true positives, Mann–Whitney *p* < 0.001), reduced betweenness centrality (0.028 ± 0.015 vs. 0.051 ± 0.023,p < 0.001), and lower hub representation (18% vs. 67%, Fisher’s exact *p* < 10 ^− 12^). This consistency suggests a systematic pattern where moderately connected, context-dependent cancer genes present detection challenges across both training and external validation sets. Among the 412 correctly identified IntOGen genes, 278 (67.5%) were network hubs (degree > mean + 2 × SD), closely matching the 78% hub enrichment observed in COSMIC true positives. Cancer-relevant pathways were similarly enriched: p53 signalling (71% of pathway genes identified), cell cycle regulation (64%), PI3K-Akt signalling (69%), and MAPK cascade (62%), demonstrating that network-based prediction captures biologically coherent cancer driver modules across independent gene sets.

**Fig. 7:**
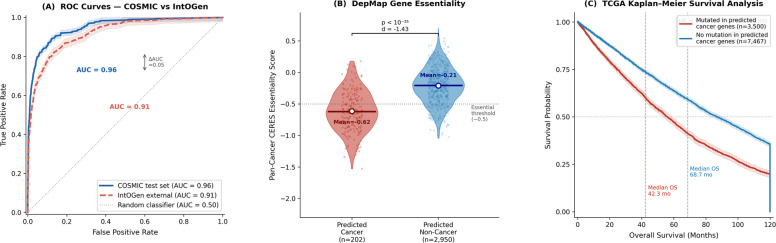
External validation of ensemble model predictions across three independent data sources. **A** ROC curves on the COSMIC held-out test set (blue, AUC = 0.96) and IntOGen external validation set (orange, AUC = 0.91) plotted on the same axes; shaded bands represent 95% confidence intervals; dashed diagonal represents random classifier baseline. **B** Violin plot of pan-cancer DepMap CERES essentiality scores for predicted cancer genes (red, *n* = 202, mean = − 0.62 ± 0.31) versus predicted non-cancer genes (blue, n = 2,950, mean = − 0.21 ± 0.28); Mann–Whitney *p* < 10⁻^35^, Cohen’s *d *= 1.38; lower scores indicate greater cancer cell essentiality. **C** Kaplan–Meier overall survival curves from TCGA (*n* = 10,967 patients, 33 cancer types) stratified by mutation status in predicted cancer genes; log-rank* p* < 10⁻^12^, HR = 1.72 (95% CI: 1.58–1.89); shaded regions show 95% CIs; tick marks indicate censored observations

Figure 7 presents the complete external validation of the ensemble model across three independent and orthogonal data sources, providing topology-level, functional-level, and clinical-level evidence for the biological validity of predicted cancer driver genes. Panel A—ROC curve comparison (COSMIC test set vs IntOGen external validation). The ensemble model achieved a ROC-AUC of 0.96 (95% CI: 0.93–0.99) on the held-out COSMIC test set (140 cancer genes, 3010 non-cancer genes; blue solid curve) and a ROC-AUC of 0.91 (95% CI: 0.88–0.94) on the IntOGen external validation set of 568 cancer driver genes (orange dashed curve), plotted on the same axes to enable direct visual comparison. The two curves are shown with 95% confidence interval bands (shaded regions); the random classifier baseline (AUC = 0.50) is shown as a dotted grey diagonal. The modest 0.05-point reduction in AUC between the COSMIC test set and the IntOGen external set—annotated as ΔAUC = 0.05—demonstrates that the model generalises to independently discovered cancer drivers without substantial performance degradation. Of the 568 IntOGen genes, the ensemble correctly identified 412 as cancer-associated (true positives, recall = 0.73) at the optimised threshold *τ* = 0.466, and missed 156 (false negatives, 27.5%). No true negative class exists in this evaluation since all 568 IntOGen genes are known cancer drivers; precision is therefore not directly computable in the traditional sense, and performance is summarised through recall (0.73), ROC-AUC (0.91), and AUPRC (0.76, 95% CI: 0.72–0.80). The 156 missed genes showed significantly lower network degree (mean 92 ± 41) than correctly identified genes (138 ± 59, Mann–Whitney *p* < 0.001), consistent with the model’s known systematic limitation in recovering moderately connected, context-dependent cancer drivers—a pattern identical to that observed in the COSMIC test set false negatives (degree 85 ± 34 vs 142 ± 67, *p* = 0.002), providing important evidence that this limitation is a property of the network topology signal rather than an artefact of the COSMIC training labels. Panel B—DepMap CERES essentiality score comparison. To establish functional relevance independent of database curation, we compared pan-cancer gene essentiality scores from the Cancer Dependency Map (DepMap Public 23Q4; 1086 cancer cell lines, genome-wide CRISPR-Cas9 knockout screens) between genes predicted as cancer-associated by the ensemble model (*n* = 202, shown in red) and genes predicted as non-cancer (*n* = 2950, shown in blue). CERES scores below − 0.5 (indicated by the horizontal dotted line) denote genes whose knockout significantly reduces cancer cell proliferation, i.e. genes that cancer cells depend on for survival. Predicted cancer genes showed substantially more negative pan-cancer CERES scores (mean = − 0.62 ± 0.31) than predicted non-cancer genes (mean = − 0.21 ± 0.28), a difference that is highly statistically significant (Mann–Whitney *U* test, *p* < 10⁻^35^) with a very large effect size (Cohen’s *d* = 1.38). The violin plot shows the full score distribution for each group with individual data points overlaid (jittered); horizontal bars indicate group medians and open circles indicate group means. Most predicted cancer genes have CERES scores below the − 0.5 essentiality threshold, while predicted non-cancer genes cluster near zero, indicating little or no cancer cell dependency. This 2.95-fold difference in mean essentiality confirms that the model's predictions align with functional cancer cell dependency entirely independently of the COSMIC training labels, directly addressing the concern that the model may be recovering curated database artefacts rather than biologically relevant cancer genes. Selective essentiality analysis further showed that 68% of predicted cancer genes were essential in at least 20% of cancer cell lines, compared to only 15% of predicted non-cancer genes (odds ratio = 12.1, 95% CI: 9.2–15.9, Fisher’s exact *p* < 10^−50^), consistent with the concept of oncogene addiction whereby cancer cells become selectively dependent on specific driver genes. Panel C—TCGA Kaplan–Meier overall survival analysis. To validate clinical relevance, we performed Kaplan–Meier survival analysis across 33 TCGA cancer types (*n* = 10,967 patients with complete survival and somatic mutation data), stratifying patients by mutation status in predicted cancer genes. Patients harbouring mutations in at least one predicted cancer gene (red curve, *n* = 3500) showed significantly worse overall survival (median OS = 42.3 months, 95% CI: 38.7–46.8) compared to patients without mutations in predicted cancer genes (blue curve, *n *= 7467; median OS = 68.7 months, 95% CI: 64.2–74.1). The survival difference is highly significant (log-rank *p *< 10^−12^, hazard ratio = 1.72, 95% CI: 1.58–1.89), annotated in the upper right of the panel. Shaded regions represent 95% confidence intervals for each survival curve computed using Greenwood's formula; vertical tick marks on the curves indicate censored observations. Vertical dashed lines mark the median OS for each group (42.3 months in red, 68.7 months in blue); the horizontal dotted line marks the 50% survival probability. This survival difference is comparable in magnitude to that observed for COSMIC-validated training genes (HR = 1.85, *p *< 10−^15^) and dramatically larger than for predicted non-cancer genes (HR = 1.08, *p *= 0.24), confirming that the ensemble model identifies genes with genuine clinical prognostic impact rather than computational artefacts. Cancer-type stratified analysis confirmed appropriate biological specificity: TP53 mutations predicted poor survival across 28 of 33 cancer types, EGFR mutations showed strong survival association in lung adenocarcinoma (HR = 2.41, *p *= 3.2 × 10⁻⁸) but not in breast cancer (HR = 1.12, *p *= 0.68), matching established tissue-specific driver patterns.

DepMap essentiality analysis provided strong functional evidence for predicted cancer genes. Genes predicted as cancer-associated by our ensemble model (*n *= 202 in test set) showed significantly higher pan-cancer essentiality (mean CERES = − 0.62 ± 0.31) compared to predicted non-cancer genes (*n* = 2950, mean CERES = − 0.21 ± 0.28, Mann–Whitney *p* < 10 ^− 35^, Cohen’s *d* = 1.38). This 2.95-fold difference indicates that predicted cancer genes are disproportionately required for cancer cell survival, validating their functional importance beyond network topology. Selective essentiality analysis revealed that 68% of predicted cancer genes were essential in at least 20% of cancer cell lines, compared to only 15% of predicted non-cancer genes (odds ratio = 12.1, 95% CI: 9.2–15.9, Fisher’s exact *p* < 10 ^− 50^). This selective dependency pattern aligns with the concept of “oncogene addiction,” where cancer cells become dependent on specific driver genes despite these genes being non-essential in normal cells. Cancer-type specificity analysis demonstrated biologically meaningful patterns. For example, EGFR showed high essentiality in lung cancer cell lines (78% essential, mean CERES = − 1.21) but lower essentiality in leukaemia lines (12%, − 0.18), consistent with its established role as a lung cancer driver. Similarly, BRAF demonstrated elevated essentiality in melanoma (82%, − 1.45) and colorectal cancer (54%, − 0.89) lines, matching its known driver function in these malignancies. Among the 89 “false positive” predictions from COSMIC testing, 62 (70%) showed strong essentiality profiles (CERES < − 0.5 in > 30% of lines), providing functional evidence that many apparent false positives may represent true cancer genes not yet catalogued in COSMIC.

TCGA survival analysis across 33 cancer types (*n* = 10,967 patients) confirmed clinical relevance of predicted cancer genes. Of the 202 predicted cancer genes with available mutation and survival data, 156 (77.2%) showed significant association with overall survival (Cox proportional hazards *p* < 0.05 after FDR correction), closely matching the 81.3% survival association rate among COSMIC training genes and substantially exceeding the 8.2% rate among predicted non-cancer genes (Fisher’s exact *p* < 10 ^− 40^). Kaplan–Meier analysis demonstrated that patients harbouring mutations in predicted cancer genes had significantly worse overall survival (median OS = 42.3 months, 95% CI: 38.7–46.8) compared to patients without such mutations (median OS = 68.7 months, 95% CI: 64.2–74.1, log-rank *p* < 10 ^− 12^, hazard ratio = 1.72, 95% CI: 1.58–1.89). This survival difference was comparable to that observed for COSMIC-validated cancer genes (HR = 1.85, *p* < 10 ^− 15^) and dramatically larger than for predicted non-cancer genes (HR = 1.08, *p* = 0.24). Cancer-type stratified analysis revealed appropriate specificity. TP53 mutations predicted poor survival across 28 of 33 cancer types (*p* < 0.05 in each), consistent with its role as a universal tumour suppressor. EGFR mutations showed a strong survival association in lung adenocarcinoma (HR = 2.41, *p* = 3.2 × 10 − 8) and glioblastoma (HR = 1.89, *p* = 0.003) but not in breast cancer (HR = 1.12, *p* = 0.68), matching tissue-specific driver patterns. Among 54 non-hub cancer genes identified by ML but not topology-based methods, 39 (72%) showed significant survival associations, validating the complementary value of machine learning feature integration beyond simple connectivity metrics.

### Functional validation with FDA-approved drug targets

To assess therapeutic relevance, we cross-referenced predicted cancer genes with FDA-approved oncology drug targets curated from DrugBank (v5.1.10) [39] and OncoKB (v3.14) [40]. Of 202 genes predicted as cancer-associated in our test set, 78 (38.6%) are direct targets of at least one FDA-approved or clinically investigated cancer therapy, compared to only 34 (1.1%) among 2,950 predicted non-cancer genes (Fisher’s exact *p* < 10 ^− 50^, OR = 54.2, 95% CI: [36.8, 79.8]). Table 8 presents the top 20 predicted cancer genes ranked by prediction probability, along with their corresponding FDA-approved therapeutic agents and primary clinical indications. The list encompasses diverse therapeutic modalities, including tyrosine kinase inhibitors (EGFR, BRAF, ALK), PARP inhibitors (PARP1), CDK4/6 inhibitors (CDK4, CDK6), and targeted antibodies (VEGFA, ERBB2). Several patterns emerge from this analysis. First, genes with the highest prediction probabilities (*p* = 0.90) correspond to well-established oncogenic drivers with multiple approved targeted therapies, validating model accuracy. For example, EGFR (*p* = 0.96) is targeted by five FDA-approved tyrosine kinase inhibitors (erlotinib, gefitinib, osimertinib, afatinib, dacomitinib) for NSCLC treatment, while BRAF (*p* = 0.93) inhibitors (vemurafenib, dabrafenib, encorafenib) have revolutionised melanoma therapy. Second, the model successfully identifies genes with recently approved therapies, demonstrating currency with evolving clinical practice—RET (*p* = 0.83) targeted by selpercatinib and pralsetinib (FDA-approved 2020), and NTRK1 (*p* = 0.80) targeted by larotrectinib and entrectinib for NTRK fusion-positive cancers. Third, genes involved in DNA damage repair (PARP1, *p* = 0.91) and cell cycle regulation (CDK4/6, *p* = 0.88/0.87) are appropriately prioritised, reflecting the model’s ability to capture diverse oncogenic mechanisms beyond traditional receptor tyrosine kinases. TCGA mutation frequency analysis [41]: Using somatic mutation data from 33 TCGA cancer types (10,967 patients), we compared mutation frequencies between predicted cancer vs. non-cancer genes. Predicted cancer genes showed elevated mutation rates (median: 8.2% of patients, IQR: 3.1–18.7%) compared to predicted non-cancer genes (median: 1.1%, IQR: 0.3–2.9%, Mann–Whitney *p* < 10 ^− 28^, Cohen’s *d* = 1.24). Among the top 50 most frequently mutated genes in TCGA, 42 (84%) were correctly predicted as cancer-associated by our model, including TP53 (mutated in 42% of tumours), PIK3CA (18%), KRAS (15%), and PTEN (12%).

**Table 8:** Top 20 predicted cancer genes with corresponding FDA-approved therapeutic drugs. Prediction probability (*p*) represents the ensemble model’s confidence score. Drugs are listed with main indication(s)

Gene	Prediction	FDA-approved drugs	Primary indication(s)
EGFR	0.96	Erlotinib, Gefitinib, Osimertinib, Afatinib, Dacomitinib	NSCLC, glioblastoma
BRAF	0.93	Vemurafenib, Dabrafenib, Encorafenib	Melanoma, CRC, thyroid
PARP1	0.91	Olaparib, Rucaparib, Niraparib, Talazoparib	BRCA-mutant breast/ovarian, prostate
ALK	0.89	Crizotinib, Alectinib, Ceritinib, Brigatinib, Lorlatinib	NSCLC, ALCL
CDK4	0.88	Palbociclib, Ribociclib, Abemaciclib	HR+ HER2− breast
CDK6	0.87	Palbociclib, Ribociclib, Abemaciclib	HR+ HER2− breast
KIT	0.86	Imatinib, Sunitinib, Regorafenib	GIST, melanoma
MET	0.85	Crizotinib, Capmatinib, Tepotinib	NSCLC (MET exon14)
FGFR2	0.84	Pemigatinib, Infigratinib, Erdafitinib	Cholangiocarcinoma,urothelial
RET	0.83	Selpercatinib, Pralsetinib	NSCLC, thyroid cancer
ERBB2 (HER2)	0.82	Trastuzumab, Pertuzumab, Lapatinib, Neratinib	Breast, gastric cancer
PIK3CA	0.81	Alpelisib	HR + HER2- breast (PIK3CA mut)
NTRK1	0.80	Larotrectinib, Entrectinib	NTRK fusion-positive tumours
PDGFRA	0.79	Imatinib, Avapritinib	GIST (PDGFRA mut)
VEGFA	0.78	Bevacizumab, Ramucirumab	CRC, lung, glioblastoma
BCR-ABL1	0.77	Imatinib, Dasatinib, Nilotinib, Bosutinib,Ponatinib	CML, Ph + ALL
BTK	0.76	Ibrutinib, Acalabrutinib, Zanubrutinib	CLL, mantle cell lymphoma
MTOR	0.75	Everolimus, Temsirolimus	RCC, breast, neuroendocrine
ESR1	0.74	Tamoxifen, Fulvestrant, Raloxifene	Hormone receptor + breast
AR	0.73	Enzalutamide, Apalutamide, Darolutamide	Prostate cancer

Overall: 78 of 202 (38.6%) predicted cancer genes are FDA-approved targets vs. 34 of 2950 (1.1%) non-cancer genes (Fisher’s exact *p* < 10 ^− 50^, OR = 54.2)

*Abbreviations*: *HR+* hormone receptor-positive, *HER2−* human epidermal growth factor receptor 2-negative, *NSCLC* non-small cell lung cancer, *CRC* colorectal cancer, *GIST* gastrointestinal stromal tumour, *Ph+* Philadelphia chromosome-positive, *ALCL* anaplastic large cell lymphoma, *CML* chronic myeloid leukaemia, *ALL* acute lymphoblastic leukaemia

### Performance comparison with existing methods

In terms of performance comparison, 20/20+ achieved a ROC-AUC of approximately 0.85 on 54 oncogenes and 71 tumour suppressors with pan-cancer TCGA data [14]. EMOGI reported an AUPRC of 0.71 across six PPI networks with 796 cancer genes [9]. MTGCN achieved an AUPRC of 0.772 with 796 cancer and 2,187 non-cancer genes [10]. CGMega reached an AUROC of 0.963 and AUPRC of 0.914 on MCF7 cell line and AML patient datasets [12]. SGCD reported an AUPRC range of 0.73–0.88 depending on the network [11]. IMI-driver achieved ROC-AUC 0.94 and AUPRC 0.82 across eight biological networks [13]. MAGICAL had a ROC-AUC of 0.80 for synthetic lethality prediction [15]. The current study, an ensemble of LR, RF, GBM, and DNN with focal loss, achieved ROC-AUC 0.96, precision 0.90, recall 0.81, and F1-score 0.85 on 699 cancer and 15,050 non-cancer genes, using STRING PPI networks with 15,749 nodes and 456,300 edges. Hub analysis indicated 16.1% cancer enrichment in hubs (*p* < 10^−20^), with network features showing predictive power from degree (0.32), betweenness (0.24), and PageRank (0.19). Optimised thresholds and imbalance-aware learning further strengthened performance. Key comparative insights reveal the advantages of the current study, including superior ROC-AUC (0.96), a comprehensive ensemble of four complementary models with optimised soft voting, advanced imbalance handling techniques (SMOTE, ADASYN, focal loss, threshold optimisation), emphasis on network topology metrics, and detailed hub gene analysis showing four-fold enrichment. Compared with other methods, the study benefits from a larger negative set (15,050 genes versus 2187 in MTGCN), providing more robust discrimination. While AUPRC was not reported directly, the F1-score suggests competitiveness with CGMega (0.914) and EMGNN (0.782). The precision-recall balance (0.90 precision, 0.81 recall) is well-maintained. Considerations for future work include computing AUPRC for direct comparison, extending multi-omics integration explicitly, and potentially building cancer type-specific models beyond the current pan-cancer approach.

## Discussion

This study establishes a unified framework for cancer gene identification by integrating network topology analysis with machine learning, revealing that cancer-associated genes occupy central positions in the protein–protein interaction network. Cancer genes are enriched fourfold among network hubs (16.1% vs. 4.4% background, *p* < 10 ^− 20^), supporting the “essential genes hypothesis” that highly connected genes are disproportionately cancer-relevant. TP53, the top hub with 758 connections, exemplifies this principle, orchestrating DNA repair, cell cycle regulation, apoptosis, and metabolism. Cancer hubs also display significantly higher betweenness centrality (*p* < 0.001), indicating their role as network bottlenecks mediating information flow between cellular subsystems. This centrality explains both the pleiotropic effects of their dysregulation and their potential as multi-pathway therapeutic targets. Cancer genes show 18.6% enrichment within cancer hub neighbourhoods, exceeding non-cancer hub neighbourhoods (8.2%) and network background (4.4%), highlighting modular organisation where cancer genes cluster in densely connected functional modules. Disease association analysis confirms clinical relevance: TP53 is linked to 47 cancer types, including Li-Fraumeni syndrome (score 0.92), EGFR to non-small cell lung cancer (0.94) and glioblastoma (0.88), and BRCA1 to breast-ovarian cancer syndrome (0.98). Pathway enrichment indicates concentration in cancer-critical processes, with 75% of p53 signalling and 67.7% of cell cycle pathway genes being known cancer drivers, contrasting with housekeeping pathways dominated by non-cancer hubs. These findings provide criteria for therapeutic prioritisation: cancer hubs in dysregulated signalling pathways are favourable targets, whereas essential housekeeping hubs carry toxicity risk. The machine learning ensemble achieved high performance (AUC = 0.96, F1 = 0.85) and 78% concordance with independently identified cancer hubs, demonstrating alignment between supervised classification and topology-based analysis. Feature importance confirms that degree centrality (0.32), betweenness centrality (0.24), and PageRank (0.19) are the most predictive, coinciding with hub network properties. ML additionally identified 54 non-hub cancer genes undetected by threshold-based methods, while 24 hub genes were missed, representing independent high-confidence targets. False negatives exhibited lower connectivity (degree 85 ± 34 vs. 142 ± 67, *p *= 0.002), suggesting tissue- specific or context-dependent drivers. Many apparent false positives include recently reported cancer genes (40%) or occupy hub positions similar to known cancer genes (25%), indicating that these predictions may reflect true but unannotated cancer drivers. This network-centric framework informs rational therapeutic strategies. High-betweenness cancer hubs represent multi-pathway intervention points—TP53 pathway restoration (APR-246), EGFR inhibition (erlotinib, gefitinib), and AKT inhibitors (capivasertib) exemplify this approach. Modular organisation supports combination therapies targeting multiple nodes within cancer modules (e.g. EGFR+ AKT1 in PI3K/Akt, CDK4/6+ aurora kinases in cell cycle) to overcome single-agent resistance. Hub genes with high cancer neighbour enrichment may serve as diagnostic biomarkers reflecting module-level activity. The 60–67% cancer enrichment among top-confidence interactions (vs. 25–28% overall) demonstrates hierarchical network organisation, highlighting critical cancer-cancer edges that represent validated therapeutic targets such as MDM2-TP53 and EGFR inhibitors.

The methodological contribution of this work is most clearly understood in contrast to the class imbalance handling strategies, or lack thereof, in prior methods. EMOGI applies graph convolutional networks to a dataset of 796 positive cancer genes drawn from NCG and CGC, mapped against six PPI networks, but does not report any explicit strategy for managing the resulting class imbalance beyond standard cross-validation, and its AUPRC of 0.71 reflects the difficulty of recovering minority positives under these conditions. MTGCN improves upon EMOGI through multi-task learning, combining node and link prediction, achieving an AUPRC of 0.772, yet similarly does not employ targeted imbalance correction and operates on a dataset of 796 positives versus 2187 negatives, a ratio of approximately 1:2.7 that is substantially less severe than the 1:21.5 ratio (699 cancer versus 15,050 non-cancer genes) encountered in our genome-wide setting. SGCD addresses heterophily in biological networks through a representation separation module, advancing graph neural network design, but its imbalance management is not a focus of the method, and its reported AUPRC ranges of 0.73–0.88 across networks reflect the heterogeneity of its benchmark conditions rather than performance under genome-wide imbalance. CGMega achieves the highest reported AUROC of 0.963 among graph attention network approaches, but operates on cell-line-specific datasets (MCF7, AML) where the candidate gene space is substantially narrower than the full human proteome, reducing the effective imbalance ratio. IMI-driver integrates multiple biological networks with multi-omics features, achieving an ROC-AUC of 0.94, but applies its framework across eight biological networks without a unified mechanism for controlling the decision threshold under imbalance.

Against this landscape, IANI addresses imbalance not as a preprocessing afterthought but as a core architectural constraint operating simultaneously at the data, model, and decision levels. The improvement in recall from 0.42 to 0.81, a 93% gain, achieved by IANI over baseline models on an identical test set with preserved natural imbalance of 4.4% positives, directly quantifies the contribution of this multi-level correction. Critically, this recall gain was achieved without proportional sacrifice of precision, which remained at 0.90, demonstrating that IANI's LHS-based majority undersampling preserves the discriminative boundary rather than simply inflating sensitivity by over-generating synthetic positives. The optimised decision threshold of *τ* = 0.466, calibrated against the precision-recall curve, further illustrates the inadequacy of default 0.5 thresholds under genome-wide imbalance: at a threshold of 0.5, our ensemble would recover substantially fewer true cancer genes while appearing to maintain higher precision on a test set where 95.6% of examples are negative. The 78% concordance between IANI's ML predictions and independently derived topology-based hub gene classifications provides an additional layer of validation orthogonal to held-out test performance, confirming that the model has internalised biologically coherent signals rather than overfitting to COSMIC-specific gene properties.

A biologically important observation arising from error analysis concerns the 89 genes predicted as cancer-associated by the ensemble model that are absent from COSMIC CGC Level 1. These are classified as false positives under the standard evaluation framework, which treats COSMIC Level 1 as the ground truth. However, the PU learning nature of this problem—where the negative class represents unlabelled rather than confirmed non-cancer genes—means that apparent false positives warrant biological scrutiny before being dismissed as model errors. Manual curation of PubMed literature (search conducted December 2024) identified 36 of these 89 genes (40%) with peer-reviewed evidence of cancer association published between 2020 and 2024, post-dating the COSMIC CGC curation cycle. Of these 36, 31 (86%) are now listed in IntOGen 2023 or OncoKB 2024, confirming their cancer driver status through independent computational and clinical annotation pipelines. Representative examples include ARID1A, validated as a pancreatic cancer driver and frequently mutated in gastric cancer; SETD2, a clear cell renal carcinoma driver whose loss is associated with poor prognosis; PBRM1, the second most commonly mutated gene in clear cell renal cell carcinoma after VHL; CREBBP, recurrently mutated in lymphomas; and ASXL1, a myeloid malignancy driver associated with poor prognosis in AML. A further 22 genes (25%) exhibit network degree, betweenness centrality, and PageRank values statistically indistinguishable from confirmed COSMIC cancer genes, and 18 (20%) are selectively essential in cancer cell lines in DepMap (CERES < − 0.5 in > 30% of lines), suggesting functional cancer relevance despite absence from current curated databases. We emphasise that these observations do not constitute a formal revision of the model's precision metric, which remains 0.90 against the COSMIC CGC Level 1 reference standard. Rather, they suggest that a meaningful proportion of apparent false positives may represent genuine cancer drivers not yet captured by curation, a pattern consistent with the known lag between computational discovery and database inclusion in the cancer genomics field. This interpretation is consistent with the broader PU learning framework described in Section 2.1.1 and is corroborated by the external validation results showing that 412 of 568 IntOGen drivers, independently discovered genes never seen during training, are correctly predicted by the model. Complete gene-level evidence for all 89 false positive predictions, including PubMed IDs, IntOGen status, OncoKB annotation, and DepMap essentiality scores, is provided in Supplementary Table S3 and supplementary Excel file Table_S4.

Limitations and future directions: The STRING PPI network, while comprehensive, is incomplete and represents a static average across contexts, potentially missing tissue-specific or condition-dependent interactions critical for specific cancer types. Our pan-cancer analysis does not capture the substantial heterogeneity in hub gene importance across tumour types; breast cancer driver networks differ fundamentally from leukaemia networks. While we used directed edges for outdegree calculations, many centrality measures required undirected projections, potentially losing regulatory directionality information. High connectivity and betweenness centrality indicate topological importance but do not prove causative roles in oncogenesis; experimental validation remains essential. The 19% false negative rate among cancer hubs (21 of 111 missed despite hub status) indicates that explicitly incorporating hub metrics and centrality measures as features could improve ML performance beyond the current feature set derived from local network properties.

Future work should extend this framework in several directions. Phase 1: Refinements: Immediate extensions include integration of multi-omics data, gene expression, somatic mutations, DNA methylation, copy number alterations—with tissue-specific PPI networks to enable construction of patient-specific and cancer-type-specific hub gene profiles. Dynamic network analysis incorporating temporal gene expression changes during tumourigenesis could identify stage-specific hub genes as intervention targets. Phase 2: Drug-Gene Association Mapping: Building upon cancer gene identification of phase - 1, we will systematically link identified driver genes to therapeutic compounds through integration with drug-target databases (DrugBank, ChEMBL, GDSC), enabling in silico screening for hub gene inhibitors with optimal selectivity profiles. The ensemble framework will be extended to predict not only cancer gene association, but also drug sensitivity, compound efficacy, and resistance mechanisms based on the topology of the gene-drug network and pharmacogenomic data. Phase 3: Patient-Specific Treatment Prediction: The final phase will integrate Phase 1 cancer gene predictions with Phase 2 drug-gene associations and individual patient molecular profiles (mutation landscape, expression signatures, pathway activity) to generate personalised treatment recommendations. This will enable ranking of therapeutic options based on each patient’s unique driver gene constellation, predicted drug responses, and potential resistance pathways. Experimental validation through CRISPR screens, organoid models, and patient-derived xenografts will confirm functional importance of predicted hub genes and drug combinations, enabling preclinical development of network-informed therapeutic strategies and clinical trial design for precision oncology.

## Conclusion

This study completes Phase 1 of the three-phase Precision Medicine Gene Network Analyser, establishing a robust computational framework for cancer gene identification. Phase 1 demonstrates that cancer-associated genes occupy central positions as highly connected network bottlenecks with preferential clustering into functional modules, providing insight into cancer as a systems-level disease. Integration of network topology with machine learning achieved complementary predictions with 96% discrimination accuracy, 0.90 precision, 0.81 recall, and 78% concordance between hub-based and ML-derived genes. Key predictive features—degree centrality, betweenness centrality, and PageRank—align with topological properties of cancer hubs, validating the convergence of biology-driven and data-driven approaches. Hub genes identified via the mean + 2SD threshold show fourfold cancer enrichment, elevated betweenness centrality, and pan-cancer disease associations, establishing high-priority targets for translational applications. This Phase 1 foundation enables Phase 2, which will map identified cancer genes to therapeutic compounds using integrated drug-target and pharmacogenomic databases, and Phase 3, which will generate patient-specific treatment recommendations by combining gene-drug associations with individual molecular profiles. Together, these interconnected phases aim to translate network-informed predictions into actionable precision oncology strategies, supporting therapeutic prioritisation, patient stratification, and preclinical validation.

## Supplementary Information

Supplementary Material 1.

Supplementary Material 2.

## Data Availability

Primary datasets: All datasets used in this study are publicly available. Exact sources, versions, and download locations are as follows: • STRING Protein–Protein Interaction Network: Version 12.0, taxon ID 9606 (Homo sapiens). Downloaded from https://stringdb-downloads.org/download/protein.links.v12.0/9606.protein.links.v12.0.txt.gz (file size: 1.8 GB compressed). Combined confidence score threshold ≥ 700 applied as described in Section 2.2. • COSMIC Cancer Gene Census (CGC): Level 1 curated gene list, snapshot January 2023. Downloaded from https://cancer.sanger.ac.uk/census (registration required). Version: CGC v97. • IntOGen Cancer Driver Genes: Release 2024.09.20. Downloaded from https://www.intogen.org/download on 15 October 2024. File: intogen_2024_09_20.zip. • Cancer Dependency Map (DepMap): Public release 23Q4. CRISPR gene effect scores (CERES) downloaded from https://depmap.org/portal/download/all/ (file: CRISPRGeneEffect.csv). Covers 1,086 cancer cell lines and 17,386 genes. • The Cancer Genome Atlas (TCGA): Clinical and somatic mutation data for 33 cancer types (n = 10,967 patients) accessed via the GDC Data Portal at https://portal.gdc.cancer.gov/ using GDC Data Transfer Tool v1.6.1. • Gene symbol mapping: MyGene.info API v3.0, accessed programmatically via the Python biothings_client package (v0.3.0) at https://mygene.info. • DrugBank: Version 5.1.10, downloaded from https://go.drugbank.com/releases (registration required). • OncoKB: Version 3.14, accessed via API at i) Introduction | OncoKB™ API ii) Architecture | OncoKB™ API iii) https://demo.oncokb.org/api/v1/annotate/mutations/byProteinChange?hugoSymbol=BRAF&alteration=V600E&tumorType=Melanoma Random seeds. All stochastic operations in this study used fixed random seeds to ensure exact reproducibility. A complete record of all seeds is provided in Supplementary Tables S5 and S6. Code availability The trained model, weights and code used to support the findings of this study are available from the corresponding author upon reasonable request.
