# Supplementary material for: Precision Medicine Gene Network Analyser: part I—cancer driver gene identification through network topology and ensemble machine learning
Source: Genomics Inform. 2026 Jun 1;24:12. doi: 10.1186/s44342-026-00074-7 (PMC13227877; doi:10.1186/s44342-026-00074-7)
Supplement: Supplementary file 1 — Supplementary Material 1. [file 44342_2026_74_MOESM1_ESM.docx]

**Precision Medicine Gene Network Analyser: Part I - Cancer Driver Gene Identification Through Network Topology and Ensemble Machine Learning**

Rashmi Siddalingappa^1*^, Showket Hussain^2^, Deepa S^3^, Pradeep Dheerendra^4^, Shivanand Gornale^5^, Muralidhara B L^6^, Gugan Kothandan^7^

^1*Department of Computer and Data Science, York St John University, United Kingdom.^

^2 Division of Molecular Diagnostics & Mol Oncology, ICMR India.^

^3 Department of Computer Science, Christ University, Bangalore, India.^

^4 School of Psychology and Neuroscience, University of Glasgow, Glasgow, United Kingdom.^

^5 Department of Computer Science, Rani Channamma University, India.^

^6 Department of Computer Science and Applications, Bangalore University, India.^

^7 Biopolymer Modelling and Protein Chemistry Laboratory, University of Madras, India.^

*Corresponding author(s). E-mail(s): [r.siddalingappa@yorksj.ac.uk;](mailto:r.siddalingappa@yorksj.ac.uk) Contributing authors: [showket.hussain@gov.in;](mailto:showket.hussain@gov.in) [sdeepa369@gmail.com;](mailto:sdeepa369@gmail.com) [pradeep.dheerendra@gmail.com;](mailto:pradeep.dheerendra@gmail.com) shivanand [gornale@yahoo.com;](mailto:gornale@yahoo.com) [murali@bub.ernet.in;](mailto:murali@bub.ernet.in) drgugank@gmail.com;

Supplementary file

**Supplementary Table S1: Complete Sample Flow from Raw Data to Final Evaluation**

| **Stage** | **Source** | **Cancer Genes (Positive)** | **Non-Cancer Genes (Negative)** | **Total Genes** | **Notes** |
| --- | --- | --- | --- | --- | --- |
| Raw COSMIC CGC Level 1 | COSMIC | 742 | — | 742 | Seed cancer driver genes |
| After STRING network mapping | COSMIC + STRING v12 | 699 | — | 699 | 43 COSMIC genes absent from network, removed |
| Full STRING network (high confidence) | STRING v12 ≥700 | — | — | 15,749 | 456,300 edges after gene symbol mapping |
| Label assignment | COSMIC + STRING | 699 | 15,050 | 15,749 | Non-COSMIC network genes = negative class (PU framework) |
| Training set (80%, stratified) | — | 559 | 11,240 | 11,799 | Stratified split, seed=42; 4.4% positive rate preserved |
| Test set (20%, stratified) | — | 140 | 3,010 | 3,150 | Held out before any preprocessing; 4.4% positive rate preserved |
| Resampled training set (SMOTE + LHS) | — | ~1,500 | ~10,000 | ~11,500 | Applied to training only; test set untouched; ~15% positive rate |
| External validation set (IntOGen) | IntOGen 2024.09.20 | 568 | — | 568 | Not in COSMIC CGC Level 1; identified post-2020; independent validation |

Note: All sample counts are exact except the resampled training set, which varies slightly across cross-validation folds due to SMOTE neighbourhood size constraints. The test set composition (140 cancer, 3,010 non-cancer, total 3,150) was fixed before any model training and was never modified or resampled.

**Supplementary Table S2: IntOGen External Validation Gene Selection Pipeline:**

| **Filter Step** | **Description** | **Genes Remaining** | **Genes Removed** | **Removal Reason** |
| --- | --- | --- | --- | --- |
| Raw IntOGen 2024.09.20 | All high-confidence driver genes | 1,042 | — | Starting set |
| Remove COSMIC CGC Level 1 overlap | Exclude training positive genes | 731 | 311 | Present in training set |
| Temporal independence filter | Exclude pre-2020 IntOGen entries | 681 | 50 | May have influenced COSMIC curation |
| STRING network mapping | Retain genes with network features | 568 | 113 | Absent from STRING PPI network |
| **Final external validation set** |  | **568** |  |  |

IntOGen version: 2024.09.20. COSMIC CGC Level 1 snapshot: January 2023. STRING version: v12, taxon ID 9606*.*

**Supplementary Table S3: Characterisation of 89 False Positive Predictions:**

| **Gene** | **Prediction Probability** | **Category** | **IntOGen 2024** | **OncoKB 2024** | **DepMap CERES** | **Key Evidence** | **PubMed ID(s)** |
| --- | --- | --- | --- | --- | --- | --- | --- |
| ARID1A | 0.88 | Literature-supported | Yes | Yes | −0.71 | Pancreatic cancer driver; mutated in 15% gastric cancer | 33723498, 38697041 |
| SETD2 | 0.91 | Literature-supported | Yes | Yes | −0.84 | ccRCC driver; poor prognosis marker | 36694082, 23444914 |
| PBRM1 | 0.84 | Literature-supported | Yes | Yes | −0.68 | 2nd most mutated gene in ccRCC after VHL | 35545605 |
| CREBBP | 0.87 | Literature-supported | Yes | Yes | −0.79 | Recurrently mutated in lymphomas; chromatin remodelling | 36958781 |
| ASXL1 | 0.82 | Literature-supported | Yes | Yes | −0.61 | Myeloid malignancy driver; poor prognosis in AML | 31068672 |
| HDAC1 | 0.79 | Topological hub | Partial | Yes | −0.88 | Mutated in 8–12% colorectal/gastric cancers | — |
| HDAC2 | 0.76 | Topological hub | Partial | Yes | −0.82 | Epigenetic regulator; colorectal cancer | — |
| CHD4 | 0.74 | Topological hub | Yes | Partial | −0.77 | Chromatin remodelling; endometrial cancer | — |
| SMARCA2 | 0.71 | Topological hub | Yes | Yes | −0.91 | Synthetic lethal with SMARCA4 in lung cancer | — |
| *[remaining 80 genes] – Refer to Supplementary Excel file Table_S4_Complete* | | | | | | |  |

Complete table with all 89 genes available as a downloadable supplementary Excel file. Categories: Literature-supported = peer-reviewed post-2020 cancer association evidence; Topological hub = network properties indistinguishable from COSMIC cancer genes; Feature-similar = within 95% CI of COSMIC gene PCA distribution; Other = no clear cancer association identified.

**Supplementary Table S5: Complete Random Seed Registry**

| **Operation** | **Library/Function** | **Seed Value** | **Location in Code** |
| --- | --- | --- | --- |
| Train-test stratified split | sklearn.model_selection.train_test_split | 42 | data_split.py, line 47 |
| SMOTE oversampling | imblearn.over_sampling.SMOTE | 123 | resampling.py, line 31 |
| ADASYN oversampling (fallback) | imblearn.over_sampling.ADASYN | 123 | resampling.py, line 45 |
| LHS-based undersampling | pyDOE2.lhs | 456 | resampling.py, line 89 |
| Stratified k-fold CV | sklearn.model_selection.StratifiedKFold | 789 | cross_validation.py, line 22 |
| Random Forest | sklearn.ensemble.RandomForestClassifier | 42 | models/rf_model.py, line 18 |
| Gradient Boosting | sklearn.ensemble.GradientBoostingClassifier | 42 | models/gbm_model.py, line 21 |
| Logistic Regression | sklearn.linear_model.LogisticRegression | 42 | models/lr_model.py, line 15 |
| DNN weight initialisation | tensorflow.random.set_seed | 42 | models/dnn_model.py, line 34 |
| Bayesian optimisation | skopt.BayesSearchCV | 42 | hyperparameter_tuning.py, line 56 |
| NumPy global seed | numpy.random.seed | 42 | utils/reproducibility.py, line 8 |
| Python global seed | random.seed | 42 | utils/reproducibility.py, line 9 |

**Computational environment.** All analyses were performed using Python 3.9.16 managed via conda 23.3.1. The complete software environment is provided as Supplementary File S1 (environment.yml for conda) and Supplementary File S2 (requirements.txt for pip). Key package versions are listed below for reference:

**Supplementary Table S6: Machine Learning Library package versions**

| **Package** | **Version** | **Purpose** |
| --- | --- | --- |
| scikit-learn | 1.2.2 | ML models, cross-validation, metrics |
| tensorflow | 2.12.0 | Deep neural network |
| keras | 2.12.0 | DNN API |
| imbalanced-learn | 0.10.1 | SMOTE, ADASYN, resampling |
| scikit-optimize | 0.9.0 | Bayesian hyperparameter optimisation |
| networkx | 2.8.8 | Graph construction and centrality metrics |
| pandas | 1.5.3 | Data manipulation |
| numpy | 1.24.3 | Numerical computation |
| scipy | 1.10.1 | Statistical tests |
| matplotlib | 3.7.1 | Visualisation |
| seaborn | 0.12.2 | Statistical visualisation |
| pyDOE2 | 1.3.0 | Latin Hypercube Sampling |
| lifelines | 0.27.4 | Survival analysis |
| biothings_client | 0.3.0 | MyGene.info API access |

Hardware: Windows 11 Pro (64-bit), Intel Core i9-13900H (2.4 GHz, 16 cores), 32 GB DDR5 RAM, NVIDIA GeForce RTX 4060 Laptop GPU (8 GB VRAM). Training time: classical models 2–5 minutes per fold; DNN 10–15 minutes per fold.

**Supplementary Table S7: CTNNB1 column with the known high-confidence partners**

| **Rank** | **TP53 Partner** | **Cancer?** | **EGFR Partner** | **Cancer?** | **CTNNB1 Partner** | **Cancer?** | **AKT1 Partner** | **Cancer?** |
| --- | --- | --- | --- | --- | --- | --- | --- | --- |
| 1 | MDM2 | Yes | ERBB2 | Yes | APC | Yes | PIK3CA | Yes |
| 2 | CDKN1A | Yes | SRC | Yes | AXIN1 | Yes | PTEN | Yes |
| 3 | ATM | Yes | GRB2 | No | TCF7L2 | No | MTOR | No |
| 4 | CHEK2 | Yes | PIK3CA | Yes | GSK3B | No | HSP90AA1 | No |
| 5 | TP63 | Yes | STAT3 | No | TP53 | Yes | PDK1 | No |
| 6 | BRCA1 | Yes | MET | Yes | EGFR | Yes | EGFR | Yes |
| 7 | RB1 | Yes | KRAS | Yes | CDH1 | No | BRCA1 | Yes |
| 8 | PTEN | Yes | AKT1 | Yes | MET | Yes | MDM2 | Yes |
| 9 | BCL2 | Yes | EGFR | Yes | CDH2 | No | HSP90AB1 | No |
| 10 | MDM4 | No | JAK2 | No | BRCA1 | Yes | RAF1 | No |
| 11 | HIPK2 | No | PDGFRA | Yes | CCND1 | No | CDKN1B | No |
| 12 | SIRT1 | No | CBL | No | LEF1 | No | TCL1A | No |
| 13 | HDAC1 | No | HSP90AA1 | No | CTNNBIP1 | No | GSK3B | No |
| 14 | PPM1D | No | SHC1 | No | EP300 | No | SRC | Yes |
| 15 | DAXX | No | GAB1 | No | CREBBP | No | TSC2 | No |
| **Total** |  | **10/15 = 66.7%** |  | **9/15 = 60.0%** |  | **6/15 = 40.0%** |  | **8/15 = 53.3%** |

Cancer status determined by COSMIC CGC Level 1 annotation. STRING combined confidence score ≥ 900 used for top-15 partner selection.
